# Hormonally Induced Hepatocellular Carcinoma in Diabetic Wild Type and Carbohydrate Responsive Element Binding Protein Knockout Mice

**DOI:** 10.3390/cells10102787

**Published:** 2021-10-18

**Authors:** Vincent Nuernberger, Sharif Mortoga, Christoph Metzendorf, Christian Burkert, Katrina Ehricke, Elisa Knuth, Jenny Zimmer, Stephan Singer, Neetika Nath, Majedul Karim, Mohd Yasser, Diego F. Calvisi, Frank Dombrowski, Silvia Ribback

**Affiliations:** 1 Institut fuer Pathologie, Universitaetsmedizin Greifswald, Friedrich-Loeffler-Str. 23e, 17475 Greifswald, Germany; vincent.nuernberger@stud.uni-greifswald.de (V.N.); sharif.mortoga@med.uni-greifswald.de (S.M.); christoph.metzendorf@igp.uu.se (C.M.); christian.burkert1@gmail.com (C.B.); katehricke@gmail.com (K.E.); elisa.knuth@gmx.de (E.K.); jenny.zimmer_privat@freenet.de (J.Z.); stephan.singer@med.uni-tuebingen.de (S.S.); smmajedul.karim@med.uni-greifswald.de (M.K.); Mohd.Yasser@med.uni-greifswald.de (M.Y.); frank.dombrowski@uni-greifswald.de (F.D.); 2 Department of Immunology, Genetics and Pathology, Uppsala University, 75108 Uppsala, Sweden; 3 Institut fuer Pathologie, Universitaetsklinikum Tübingen, 72076 Tübingen, Germany; 4 Institut fuer Bioinformatik, Universitaetsmedizin Greifswald, 17475 Greifswald, Germany; neetika.nath@uni-greifswald.de; 5 Institut fuer Pathologie, Universitaetsklinikum Regensburg, 93053 Regensburg, Germany; Diego.Calvisi@klinik.uni-regensburg.de

**Keywords:** hepatocarcinogenesis, hepatocellular carcinoma, ChREBP, PI3K/AKT/mTOR, intraportal pancreatic islet transplantation, clear cell foci of altered hepatocytes, preneoplastic foci

## Abstract

Objective: In the rat, the pancreatic islet transplantation model is an established method to induce hepatocellular carcinomas (HCC), due to insulin-mediated metabolic and molecular alterations like increased glycolysis and de novo lipogenesis and the oncogenic AKT/mTOR pathway including upregulation of the transcription factor Carbohydrate-response element-binding protein (ChREBP). ChREBP could therefore represent an essential oncogenic co-factor during hormonally induced hepatocarcinogenesis. Methods: Pancreatic islet transplantation was implemented in diabetic C57Bl/6J (wild type, WT) and ChREBP-knockout (KO) mice for 6 and 12 months. Liver tissue was examined using histology, immunohistochemistry, electron microscopy and Western blot analysis. Finally, we performed NGS-based transcriptome analysis between WT and KO liver tumor tissues. Results: Three hepatocellular carcinomas were detectable after 6 and 12 months in diabetic transplanted WT mice, but only one in a KO mouse after 12 months. Pre-neoplastic clear cell foci (CCF) were also present in liver acini downstream of the islets in WT and KO mice. In KO tumors, glycolysis, de novo lipogenesis and AKT/mTOR signalling were strongly downregulated compared to WT lesions. Extrafocal liver tissue of diabetic, transplanted KO mice revealed less glycogen storage and proliferative activity than WT mice. From transcriptome analysis, we identified a set of transcripts pertaining to metabolic, oncogenic and immunogenic pathways that are differentially expressed between tumors of WT and KO mice. Of 315 metabolism-associated genes, we observed 199 genes that displayed upregulation in the tumor of WT mice, whereas 116 transcripts showed their downregulated expression in KO mice tumor. Conclusions: The pancreatic islet transplantation model is a suitable method to study hormonally induced hepatocarcinogenesis also in mice, allowing combination with gene knockout models. Our data indicate that deletion of ChREBP delays insulin-induced hepatocarcinogenesis, suggesting a combined oncogenic and lipogenic function of ChREBP along AKT/mTOR-mediated proliferation of hepatocytes and induction of hepatocellular carcinoma.

## 1. Background

### 1.1. Preneoplastic and Neoplastic Hepatocellular Lesions in Rodent Models and Humans

Hepatocellular carcinoma (HCC) is the fourth most common cancer worldwide and is associated with a poor prognosis [1,2]. Alongside the well-known carcinogenesis related to alcohol abuse or hepatitis B or C virus infection with occurrence of cirrhosis and dysplastic nodules, epidemiologic studies have also revealed an increasing association to risk factors like obesity and/or type 2 diabetes mellitus as part of the metabolic syndrome [3]. These metabolic disorders often lead to Non-Alcoholic Fatty Liver Disease (NAFLD), Non-Alcoholic Steatohepatitis (NASH), and to HCC without development of cirrhosis in about 15% of all HCC cases [4,5]. This carcinogenic process is poorly understood in humans to date.

In different animal models of hepatocarcinogenesis, preneoplastic liver lesions often progress to hepatocellular adenomas (HCA) and HCC without pre-existing cirrhosis. Among these preneoplastic lesions, clear cell foci (CCF) of altered hepatocytes are the earliest and the most frequent type [6]. These lesions are characterized by an increased proliferative activity and glycogen and fat storage due to metabolic alterations such as increased glycolysis and de novo lipogenesis, which represent insulin-mimetic effects on hepatocytes [6,7]. The intraportal pancreatic islet transplantation (IPIT) is a hormonal model to induce CCF, which has been investigated in detail in diabetic rats [8,9,10,11]. Due to the insulin-mimetic effects, CCF developed already after a few days and progressed to hepatocellular adenomas and carcinomas [10,11].

The local hyperinsulinism and simultaneous hyperglycemia due to diabetes activate the phosphoinositide-3-kinase/V-akt murine thymoma viral oncogene homolog/mammalian target of Rapamycin (PI3K/AKT/mTOR) and Rat sarcoma/mitogen activated protein kinase (Ras/MAPK) proto oncogenic pathways in the hepatocytes of the liver acini downstream of the transplanted islets [9,12]. These alterations lead particularly to reduced gluconeogenesis, increased glycolysis, and increased pentose phosphate pathway, which characterize a glycogenotic phenotype [6]. At the same time, de novo lipogenesis is also activated, promoting the lipogenic phenotype [13]. These metabolic changes, i.e., increased glycolysis, are observed in many malignant tumors, including human HCC, and are associated with a poor prognosis. The switch from a glycogenotic to a lipogenic phenotype in CCF is assumed to be an important step to malignancy [13].

CCF have also been characterized in human cirrhotic and non-cirrhotic livers by the presence of a lipogenic phenotype and activation of the PI3K/AKT/mTOR and Ras/MAPK pathways suggesting a preneoplastic nature of CCF also in humans [12,14].

### 1.2. Role of Carbohydrate-Response Element-Binding Protein in Hepatocellular Metabolism and Carcinogenesis

Carbohydrate-response element-binding protein (ChREBP) is an important transcription factor related to the PI3K/AKT/mTOR pathway [12,15]. Previous investigations indicated its proto-oncogenic potential, especially mediated by AKT/mTOR, but also related to cMyc and/or p53-signalling [16,17]. ChREBP is a glucose-dependent transcription factor, which binds to the carbohydrate responsive element (ChoRE) motifs in gene promoters [18,19,20]. ChREBP target genes mainly regulate de novo lipogenesis, glycolysis, gluconeogenesis and the pentose phosphate shunt in the liver [17]. Consequently, it is a pivotal regulator of hepatocellular glucose and lipid metabolism, besides sterol regulatory element-binding protein 1 and 2 [21]. 

Recently, our group successfully transferred the IPIT model from the rat to the mouse and combined it with a knockout model of ChREBP [15].

## 2. Methods

In this study, all animals received humane care according to the criteria outlined in the “Guide for the Care and Use of Laboratory Animals”, prepared by the National Academy of Sciences and published by the National Institutes of Health (NIH publication 86-23 revised 1985). Animal experiments were approved by the Animal Policy and Welfare Committee of the Universitaetsmedizin Greifswald, Germany (LALLF-MV Rostock, Germany, ref. no. 7221.3-1-012/16; 4 May 2016). Housing of the animals was in accordance with the guidelines of the Society for Laboratory Animal Service and the German Animal Protection Law.

Highly inbred 9-week-old male C57Bl/6J wild type (WT, ChREBP^+/+^) and ChREBP-knockout (ChREBP-KO, ChREBP^−/−^) mice (*n* = 320, body weight >20 g; Charles River Laboratories, Sulzfeld, Germany) were assigned to 16 groups (see Table 1). Induction of diabetes with streptozotocin, intraportal pancreatic islet transplantation, and application of the nucleoside analog 5-Bromo-2′-deoxyuridine (BrdU) and tissue processing were applied as previously reported [15], and are described in detail in Supplementary Materials section.

### 2.1. Immunohistochemistry

Formalin-fixed and paraffin-embedded or cryopreserved, respectively, serial liver sections with 1–2 µm thickness were stained for aldolase, hexokinase II, pyruvate kinase M2 (PKM2), phosphorylated/activated AKT (pAKT), mammalian target of rapamycin (mTOR), phosphorylated/activated ribosomal protein S6 (pRPS6), acetyl-CoA carboxylase (ACAC), fatty acid synthase (FASN), glucose transporter 4 (GLUT4), phosphofructokinase (PFKL), glycogen synthase kinase 3β (GSK-3β) and BrdU.

The immunohistochemical reactions were assessed semi-quantitatively by comparing intensity in CCF or tumor with corresponding surrounding unaltered liver tissue. Negative controls were stained without any primary antibody. 

All primary antibodies with detailed information are listed in Supplementary Table S1.

### 2.2. Morphologic and Proliferation Kinetic Investigations

CCF correspond to lesions of enlarged hepatocytes with pale cytoplasm in hematoxylin and eosin (H&E) staining according to extensive glycogen storage in PAS reaction. In WT mice, hepatocytes also revealed many lipid droplets. 

HCA and HCC were identified in the liver macroscopically as space occupying lesions. HCA were diagnosed as sharply limited lesions that compressed the surrounding liver parenchyma. On the other hand, HCCs were defined by lesions with trabeculae thicker than three cell layers, revealing high numbers of mitotic figures, and a diameter of larger than 3 mm. BrdU labelling indices of CCF and unaltered liver tissue were evaluated in representative sections of mice liver tissues. All hepatocytes of CCF and 2000 hepatocytes in extrafocal tissue (EFT) were counted. Levels of proliferation were expressed based on BrdU Labelling Index (BrdU-LI, number of positive hepatocytes/total number of hepatocytes × 100%).

### 2.3. Glycogen Quantification (Automated PAS-Quantification)

Paraffin-embedded liver sections were stained by the PAS reaction and counterstained with haematoxylin in a Leica immunostainer. Sections were visually evaluated and imaged on a DMRB microscope (Leica, Wetzlar, Germany) using a DS-Fi1 digital camera (Nikon Instruments, Düsseldorf, Germany) controlled by the NIS Elements BR software. Following automated PAS-quantification is described in detail in Supplementary material and methods. 

Of 34 liver samples with PAS intensities covering the full range from weakly stained to strongly stained, glycogen content was determined biochemically using the Glycogen Assay Kit II (ab169558; Abcam, Cambridge, UK), according to the manufacturers protocol to measure glycogen.

### 2.4. Ultrastructural Analysis

Specimens of 2 mm^3^ liver tissue with and without CCF/tumors were fixed in 2.5% glutaraldehyde and embedded in glycid ether. The pieces were cut with a diamond knife in a Leica ultratome Leica EM UC7 (Leica Biosystems, Wetzlar, Germany). These 500- and 750-nm-thick semi-thin slides were stained with H&E, PAS and according to Richardson. Ultrathin sections of 70–90 nm were stained with uranyl acetate and eventually were examined with a Libra 120 electron microscope (Carl Zeiss, Jena, Germany).

### 2.5. RNA Extraction, Quality Control and RNA-seq Transcriptomic Data Analysis

Total RNA extraction from archived snap-frozen experimental mice tissues (tumor and normal liver) was performed using RNA purification kit from Macherey-Nagel (MACHEREY-NAGEL GmbH & Co. KG, Dueren, Germany) following manufacturer’s recommended protocol. Quantity of RNA was measured using a Nanodrop 8000 (Thermo Scientific, Waltham, MA, USA). Quality and integrity of RNA was assessed using a Bioanalyzer (Agilent 2100 Bioanalyzer, Agilent Technologies, Santa Clara, CA, USA). Samples with RNA Integrity Number (RIN) values more than 7 except for two samples (RIN value: 6.7 and 6.8) were processed further (Supplementary Table S2).

RNA seq analysis was performed by Genewiz NGS laboratory (Genewiz, Leipzig, Germany) using paired-end single-indexed sequencing on an Illumina Novaseq 6000 sequencing system (Illumina, Inc., San Diego, CA, USA). To begin with, sequence reads were trimmed to eliminate possible adapter sequences and nucleotides with poor quality using Trimmomatic v.0.36. Following this, the trimmed reads were mapped to the mouse reference genome (Mus musculus GRCm38) available on ENSEMBL using the STAR aligner (v.2.5.2b). Conversion of Ensemble IDs into gene symbols was performed using “EnsDb.Mmusculus.v79” (DOI: 10.18129/B9.bioc.EnsDb.Mmusculus.v79) and “org.Mm.eg.db’’ (10.18129/B9.bioc.org.Mm.eg.db). Read counts of unique genes were converted into CPM (Counts Per Million) using R/Bioconductor packages (https://combine-australia.github.io/RNAseq-R/06-rnaseq-day1.html, that include clusterProfiler v4.0.5, org.Mm.eg.db v3.13.0, EnhancedVolcano v1.11.1, VennDiagram v1.6.20, ggplot2 v3.3.5 and the accession date is 21 September 2021), and genes with CPM < 0.5 were eliminated from further analysis. The downstream differential expression analysis was performed using Fisher’s exact test as described [22]. Multiple testing correction was computed to extract adjusted *p* values using false discovery rate (FDR), Benjamini and Hochberg’s method, and Holm–Bonferroni, where False Discovery Rate (FDR) < 0.05 was considered as statistically significant. Heatmaps were generated and expressed as log_2_ fold-change between two conditions. All statistical analyses and plots were generated using data analysis software R (v4.0.4).

### 2.6. Quantitative Real-Time Polymerase Chain Reaction (qRT-PCR)

cDNA were synthesized from the same purified RNA used for RNA-seq experiments by using cDNA synthesis kit ( Thermo Fisher Scientific, Waltham, MA, USA as per the manufacturer’s instruction. SYBR-based real time quantitative PCR was performed in a Corbett Rotor-Gene 6000 real-time PCR cycler (Qiagen, Hilden, Germany) by using the SensiFAST^TM^ SYBR No-ROX Kit ( Bioline, London, UK) with respective forward and reverse primers, and the relative values of gene expression were normalized to 18S rRNA housekeeping gene. All amplifications were performed independently two times, and each time in triplicate with non-template control (NTC). The sequences of the primers used are as follows: Slc2a3, F: CCGCTTCTCATCTCCATTGTCC, R: CCTGCTCCAATCGTGGCATAGA; Slc2a6, F: GGCTCCTATCTGTGCTGATTGC, R: CCTTGGCACAAACTGGACGTAG; Pfkb4, F: GAGCCAGATGAAGAGGACGATC, R: GCAAACTCCAGCGGGTAGTGAT; Fabp7, F: CAGTCAGGAAGGTGGCAAAGTG, R: GCTTGTCTCCATCCAACCGAAC; Mycl, F: CACTCCTAGTCTGGAAGCCAGT, R: CGGTCACCACGTCAATCTCTTC; and 18S, (Mm_Rn18s_3_SG QuantiTect Primer Assay, purchased from Qiagen). Relative gene expression from real-time PCR data was analysed by using the comparative C_T_ method (also referred to as the 2^−ΔΔCT^ method) as described by Schmittgen et al. [23].

### 2.7. Statistical Analysis

All statistical analyses were performed either with R or GraphPad Prism 6.0. Quantitative data are expressed as mean ± standard error of the mean (S.E.M.).

Differences in body weight, blood glucose level, glycogen storage, diameter of CCF and tumor, proliferative activity and biochemical assays (serum ALT and AST level) were assessed using Student’s t test of normally distributed data, otherwise Wilcoxon Mann–Whitney U test was applied. Normal distribution was tested using the Shapiro–Wilk test. 

Fisher’s exact test was used for testing differences of frequency. Linear regression was tested using adjusted determination coefficient R^2^. Differences were considered significant if *** *p* < 0.001, ** *p* < 0.01, and * *p* < 0.05, and ‘‘n.s.’’ indicates not significant.

## 3. Results

Streptozotocin-induced diabetic C57Bl/6J wild type mice (WT) and ChREBP-knockout mice (KO) received an intraportal transplantation of isolated, isologous pancreatic islets into the liver. Clear cell foci, hepatocellular adenomas and carcinomas, proliferative activity, hepatocellular glycogen storage, blood glucose levels, and body weight were compared between these two strains. 

### 3.1. Hormonally Induced Hepatocarcinogenesis Leads to CCF of Altered Hepatocytes

CCF of altered hepatocytes were detectable in liver acini downstream of the transplanted islets in diabetic transplanted WT as well as ChREBP-KO mice after 6 and 12 months. Frequency of CCF did not differ between WT and KO mice after six months (WT: 8/36, 22.22%; KO: 8/18, 44.44%, n.s.).

#### 3.1.1. ChREBP Is Associated with Distinct Morphological Alterations

**Figure 1:**
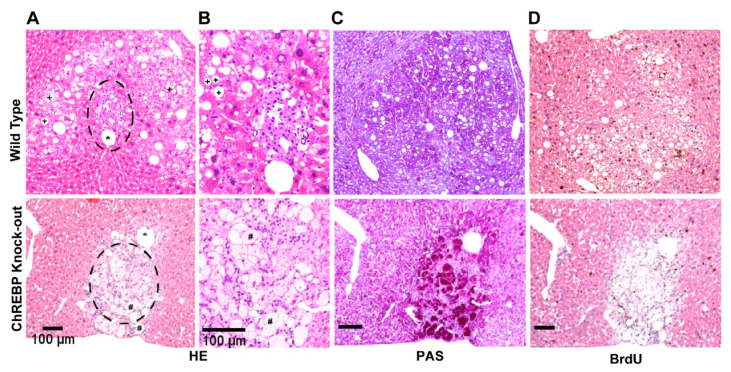
WT and KO CCF display distinct morphological alterations. Representative histological and immunohistochemical images showing CCF of altered hepatocytes in wild type (upper panel) and ChREBP-knockout (lower panel) mice after six months. CCF in WT mice revealed lipid droplets (indicated by ‘+’ symbol), which were instead lacking in CCF from KO mice. A transplanted pancreatic islet located in the middle of the WT CCF is illustrated with dashed circle (**A**) and (**B**) designates a typical CCF that corresponds to high PAS reactivity. Asterisk (*) represents portal vein branch, and hash symbols (#) indicate enlarged and swollen hepatocytes (**A**,**B**). PAS reaction was stronger in KO-CCF than in WT-CCF (**C**). Proliferative activity, as assessed by BrdU-LI, was markedly higher in CCF of WT mice compared to KO mice (**D**). Length of the lower edge (0.8 mm) (**A**–**D**). Higher magnification (0.3 mm) (**B**).

To study the underpinning role of ChREBP in CCF formation and thus in morphological alterations, we compared CCF between wild type and knock-out mice, and found distinct morphological appearances. Hepatocytes in WT-CCF revealed a pale cytoplasm and many lipid vacuoles shown by H&E staining (Figure 1A,B). The hepatocytes were not significantly enlarged. Similarly, inflammatory alterations were not detectable. As anticipated, the transplanted pancreatic islets were evident in the neighbouring portal vein branches (Figure 1A,B). The PAS reaction was slightly stronger in the cytoplasm compared to the extrafocal liver tissue. Conversely, hepatocytes of KO-CCF mice revealed massive glycogen but almost no lipid storage, suggesting inhibition of glycolysis in absence of ChREBP, and that reduction in glucose metabolism leads to glycogen accumulation in the liver (Figure 1C) [24]. Consequently, hepatocytes in CCF of KO mice appeared swollen and enlarged (Figure 1A,B). CCF in KO mice were accompanied by some inflammatory alterations with infiltrating leukocytes. Extrafocal tissues, on the other hand, did not demonstrate any detectable signs of inflammation and/or cirrhosis both in wild type and knock-out mice (supplementary Figure S11).

KO-CCF were significantly smaller than CCF in WT mice (diameter (mean ± S.E.M.): KO-CCF 392 ± 37 µm (*n* = 12) vs. WT-CCF 786 ± 119 µm (*n* = 8); *p* < 0.05). On the contrary, glycogen storage was remarkably higher in KO-CCF than in WT-CCF (63.5 ± 5.8% vs. 25.6 ± 7.0%; *p* < 0.01) (supplementary Figure S2).

#### 3.1.2. Proliferative Activity

To determine if CCF exert a proliferative effect on HCC, we next analysed the proliferation profile of CCF in both KO and WT mice. The proliferative activity, measured as BrdU Labelling Index (BrdU-LI) for one week, in CCF of WT mice was 29.04 ± 11.97% (mean ± S.E.M.), while in the surrounding EFT was 9.97 ± 1.66% (*n* = 5; n.s.). In KO-CCF, proliferative activity was higher (19.72 ± 1.67%) than in the extrafocal liver tissue (EFT; 4.42 ± 0.79%; *n* = 4; *p* < 0.001).

**Figure 2:**
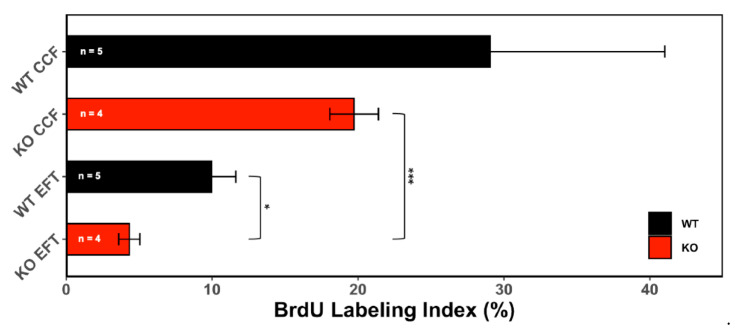
CCF mediate abnormal proliferation activity of hepatocytes. Shown data represent the proliferative activity (measured by BrdU-LI, one-week measurement via an osmotic mini pump) of CCF and extrafocal tissue (EFT) in wild type (WT) and ChREBP-knockout mice (KO). Data are depicted as mean ± S.E.M.; * *p* < 0.05; *** *p* < 0.001.

There was no difference between CCF in WT and KO mice, but proliferative activity of the EFT differed significantly (*p* < 0.05; Figure 2). 

### 3.2. CCF Signature Leads to Hepatocellular Adenomas (HCAs) and Carcinomas (HCCs)

Next, we assessed whether the activated form of CCF signature leads to HCC formation in IPIT transplanted WT mice and absence of ChREBP has a delayed effect in tumor progression. While three HCCs were already developed in diabetic transplanted WT mice (frequency 3/69; 4.44%) after 6 and 12 months, only one carcinoma in a diabetic transplanted KO mouse (frequency 1/30; 3.33%; n.s.) after 12 months was formed. This supports the notion that ChREBP deletion mitigates the tumorigenic potential in diabetic transplanted KO mice. Furthermore, four spontaneous HCAs were detectable in diabetic WT control mice following 6 and 12 months (frequency 4/33; 12.12%), whereas one HCA in non-diabetic KO control mouse and two HCAs in diabetic transplanted KO mice after 12 months were observed (frequency 3/30; 10.00%; n.s.). 

#### 3.2.1. HCAs and HCCs Are Associated with Distinct Morphological Alterations

**Figure 3:**
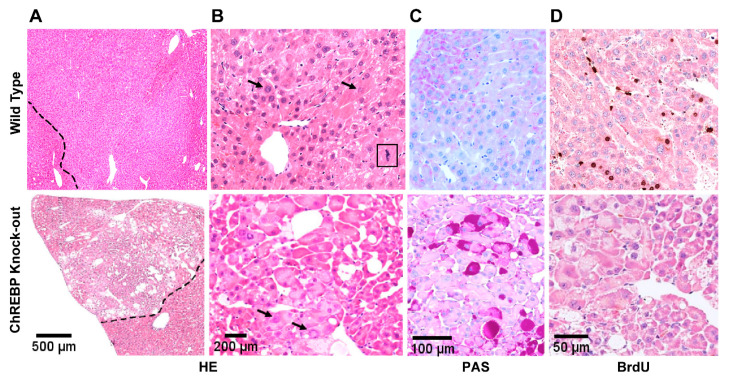
HCAs of WT and KO mice exhibit distinctive cell morphology. Representative histological and immunohistochemical images illustrate HCAs in a diabetic wild type (upper panel) and diabetic transplanted ChREBP-knockout mouse (lower panel). HCAs are featured with slightly enlarged nuclei of hepatocytes (indicated by arrows) in H&E staining (**A**,**B**), often less but partially in KO mice with increased glycogen storage in PAS reaction (**C**, lower panel) and increased proliferative activity measured by staining of proliferation marker, BrdU (**D**, lower panel) in KO mice. A clear demarcation (indicated by dotted lines) between adenomas and neighbouring extra focal liver tissues is illustrated (**A**,**B**). Square box depicts a mitotic figure (**B**, upper panel). Length of the lower edge: (**A**) 2.5 mm, (**B**) 0.4 mm, (**C**) 0.35 mm, (**D**) 0.25 mm.

**Figure 4:**
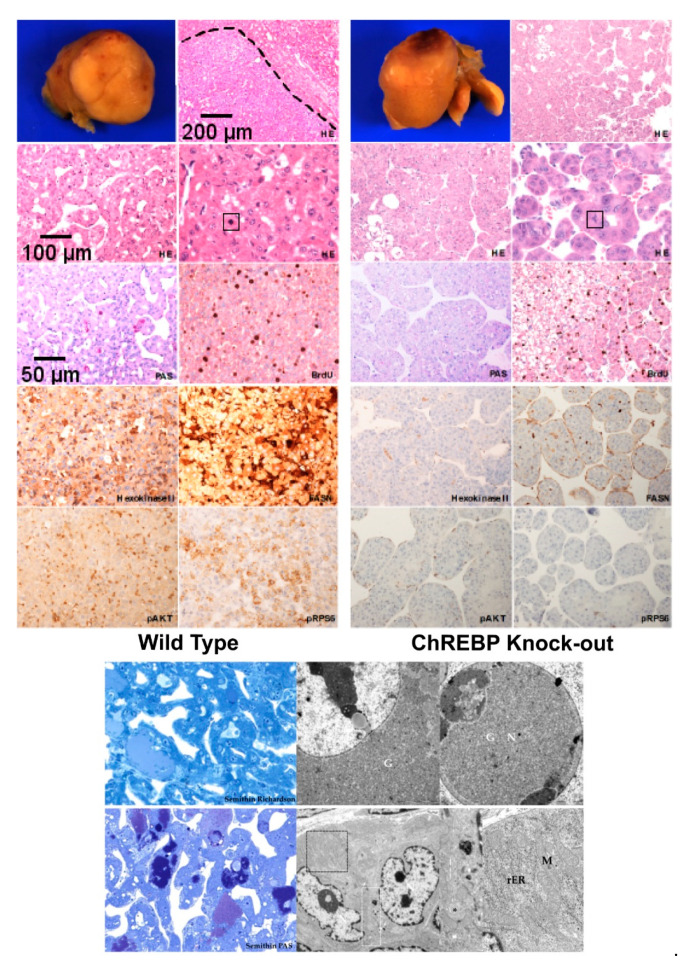
HCCs of WT mice are associated with enhanced glycolytic, de novo lipogenesis and AKT/mTOR pathway activities.

The process of hepatic tumorigenesis is a sequential event where evolution of normal epithelial cells to HCC formation is usually followed, at first, by initial formation of adenomas then transforming to fibrosis and cirrhosis, which finally progress to HCCs. Since we had revealed the formation of HCC in both wild and knock-out mice, we further inquired as to whether absence of ChREBP would show distinct, if any, changes in the morphology of the tumor. To this end, we performed H&E staining in tumor samples obtained from both experimental mice groups. Representative images shown in Figure 4 indicate HCAs with its prominent feature as mass forming tumors at the surface of livers with a diameter of 3.9 ± 1.2 mm in WT and 1.2 ± 0.2 mm in KO mice (mean ± S.E.M.; n.s.). They were distinguished from CCF with respect to their mild expansive growth without invasion of the normal parenchyma, slightly enlarged nuclei and a higher proliferative activity (Figure 3). 

HCCs were also detectable macroscopically with a mean diameter of 5.6 ± 1.2 mm in WT and 8.8 mm in KO mice (n.s.). These tumors sprouted strongly, displaced the surrounding tissue, and revealed haemorrhage and central necrosis. 

Histologically, these carcinomas were composed of optically distinct basophilic enlarged hepatocytes with solid trabecular growth with trabeculae thicker than three cell layers, augmented nuclei and prominent nucleoli, mitotic figures and necrosis, thus exemplifying prominent alterations comparable to hepatocellular malignancy. As expected, HCCs also contained PAS positive clear cell areas corresponding to cytoplasmic glycogen storage (Figure 4, lower panel). HCCs of KO mice did not differ morphologically from WT mice (Figure 4).

Upper panel: Hepatocellular carcinomas (HCC) in WT and KO mice, characterized by basophilic cytoplasm, enlarged nuclei and some mitosis (indicated by square box) in H&E staining. A few tumor cells were PAS positive. Elevated proliferative activity (BrdU) in both types of tumors was evident. Levels of glycolysis (i.e., hexokinase II), de novo lipogenesis (i.e., fatty acid synthase, FASN) and the AKT/mTOR pathway (i.e., pAKT, pRPS6), were significantly lower in KO-HCC in comparison to WT-HCC. A clear boundary depicted by broken lines distinguishes tumor tissue from healthy neighbouring liver tissues. Length of the lower edge: H&E upper right, 1.0 mm; H&E lower left, 0.5 mm; H&E lower right, PAS and immunohistochemistry, 0.25 mm.

Lower panel: Ultrastructure of hepatocellular carcinomas of diabetic transplanted mice (semithin sections, stained with the Richardson’s method and PAS and corresponding ultrathin sections). Representative electron micrographs showing atypical hepatocytes with enlarged and bizarre nuclei (N) and prominent nucleoli, small bile canaliculus (*) between hepatocytes, sometimes with increased glycogen (G) storage with α-particles in the cytoplasm and also in nuclei, sometimes with glycogen-poor cytoplasm and augmented rough endoplasmic reticulum (rER) and mitochondria (M). 

By employing transmissive electron microscopy, and thus examining ultrathin tissue sections at ultrastructural level, atypical hepatocytes of HCCs revealed distinct enlarged and bizarre nuclei with prominent nucleoli accompanied by an increased glycogen storage with α-particles in the cytoplasm. In hepatocytes, glycogen-poor cytoplasm and augmented endoplasmic reticulum (ER) and mitochondria in some instances were also visible. Notably, ultrastructural morphology of HCCs did not differ between genotypes (Figure 4, lower panel). It suggests that ChREBP does not have any marked additional effects on the morphological alterations pertinent to hepatocarcinogenesis.

#### 3.2.2. Immunohistochemical Expression Patterns of Glycolytic, Lipogenic and Molecular Pathways

To investigate the molecular pathways promoting glycolysis, de novo lipogenesis and AKT/mTOR pathway, we performed immunohistochemical staining of certain crucial enzymes involved in these particular pathways. HCCs of WT mice revealed an upregulation of enzymes of glycolysis (i.e., glucose transporter 4, hexokinase II, aldolase, phosphofructokinase, and pyruvate kinase), de novo lipogenesis (i.e., fatty acid synthase, acetyl-CoA carboxylase) and of the AKT/mTOR pathway (i.e., p-AKT, p-mTOR, p-RPS6, p-GSK-3β). Surprisinlgy, levels of these metabolic and molecular pathways were markedly lower in KO-HCC (Figure 4*,* upper panel). These data in aggregate suggest that ChREBP regulates oscillating activity of the deregulated metabolic pathways associated with the hepatocarcinogenic process and its absence significantly abrogates their aberrant activation.

### 3.3. Glycogen Storage in Unaltered Liver Tissue

**Figure 5:**
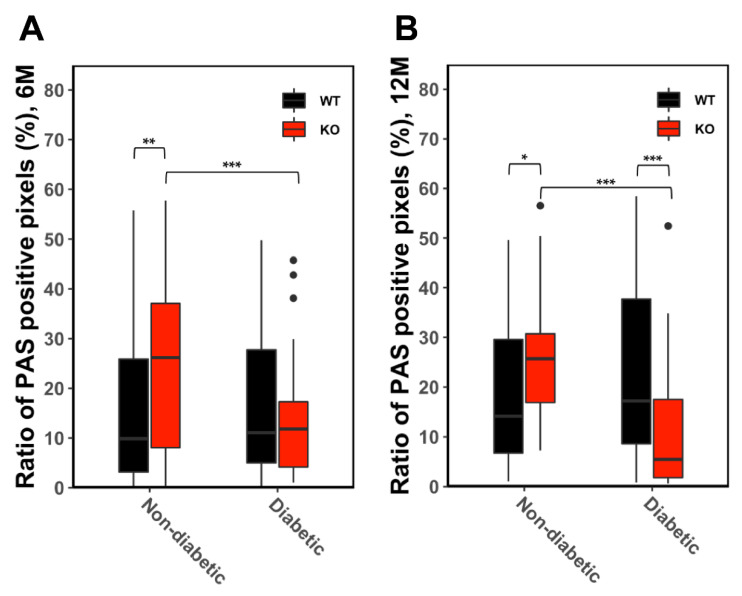
Glycogen content in the extrafocal liver tissue. Illustrative box plots represent glycogen content as PAS positive proportion of the liver section after six months (**A**). Hepatocytes of non-diabetic wild type (WT) mice stored significantly less glycogen than ChREBP-knockout (KO) mice. Non-diabetic KO-mice stored significantly more glycogen than diabetic KO mice. After 12 months (**B**), non-diabetic wild type mice (WT) also stored significantly less glycogen than ChREBP-knockout mice (KO). Hepatocytes of diabetic WT mice revealed significantly more glycogen than diabetic KO mice. Non-diabetic KO-mice stored significantly more glycogen than diabetic KO mice. * *p* < 0.05; ** *p* < 0.01; *** *p* < 0.001.

Next, we assessed the glycogen accumulation, and morphometrical analysis of PAS reaction yielded differences in glycogen storage in the liver parenchyma of diabetic and non-diabetic mice. Hepatocytes of non-diabetic KO mice contained significantly more glycogen than diabetic KO mice after 6 and 12 months. In WT mice, glycogen storage was not altered after diabetes induction. In WT diabetic mice, hepatocytes contained more glycogen than diabetic KO mice after 12 months. On the other hand, non-diabetic WT mice exhibited lower glycogen than non-diabetic KO mice after 6 and 12 months (Figure 5). 

### 3.4. Proliferative Activity of Unaltered Liver Tissue

To assess the differential regulation of hepatocyte proliferation in the unaltered extrafocal liver tissue, DNA synthesis was measured by immunohistochemical staining of BrdU incorporation between WT and KO mice. As expected, BrdU-LI of hepatocytes in the unaltered extrafocal liver tissue was lower in diabetic transplanted KO than in WT mice following 6 and 12 months (Table 2). As shown in supplementary Figure S3, there were further differences in each strain. Notably, diabetic transplanted WT mice displayed a stronger proliferative activity than control mice after 6 and 12 months. However, diabetic transplanted KO mice had only a higher proliferative activity than diabetic and non-diabetic control mice after 6 months. 

### 3.5. Blood Glucose Level, Body Weight, and Serum ALT and AST Levels

Finally, we measured the blood glucose level and body weight. Blood glucose level of both genotypes differed only in the diabetic groups. Indeed, diabetic transplanted KO mice had a higher blood glucose level than diabetic transplanted WT mice after 6 months (26.2 ± 0.4 mmol/L (*n* = 18) vs. 20.4 ± 0.6 mmol/L (*n* = 36) (mean ± S.E.M.); *p* < 0.001), as well as diabetic WT and KO mice after 12 months ((26.0 ± 0.7 mmol/L (*n* = 13) vs. 21.0 ± 1.2 mmol/L (*n* = 13); *p* < 0.01)). Mean blood glucose levels in non-diabetic WT and KO mice did not differ (supplementary Figure S4). Noticeably, there was no difference between diabetic mice transplanted or not with pancreatic islet.

Body weight was generally lower in diabetic than in non-diabetic mice and tended to be higher in WT mice after 12 months than after 6 months in diabetic as well as in non-diabetic WT mice. Diabetic transplanted WT mice had a significantly higher body weight compared to diabetic transplanted KO mice after 6 and 12 months (supplementary Figure S5).

Similarly, we assessed the serum alanine aminotransferase (ALT) and aspartate aminotransferase (AST) level in non-diabetic control and diabetic transplanted mice. Data presented in supplementary Figure S10 indicate an increased level in both ALT and AST in diabetic (WT and KO) mice compared to corresponding non-diabetic control mice. Diabetic KO mice exhibited an increase in the levels of ALT and AST after 6 months, whereas after 12 months, a reverse trend was noticed. Notably, no statistical significance between the comparisons was observed. 

### 3.6. Transcriptional Profiling of Liver Tissues (Tumor and Extrafocal Liver Tissue) to Characterize Prominent Dysregulated Genes

To examine ChREBP-dependent response on metabolic processes and thus on hepatocarcinogenesis progression, we next performed RNA sequencing-based transcriptional profiling on tissues derived from liver tumor (tumor formed in 12 months) of knock-out as well as corresponding wild type mice, and compared them in between and also with the non-tumorous control tissue obtained from same mouse liver. Of note, both groups of mice were diabetic and pancreatic islet transplanted. Moreover, non-diabetic and non-transplanted liver tissues obtained from corresponding mice were also included in the analysis. An elaborate categorization and approach followed for comparison between groups is illustrated in supplementary Figure S6.

**Figure 6:**
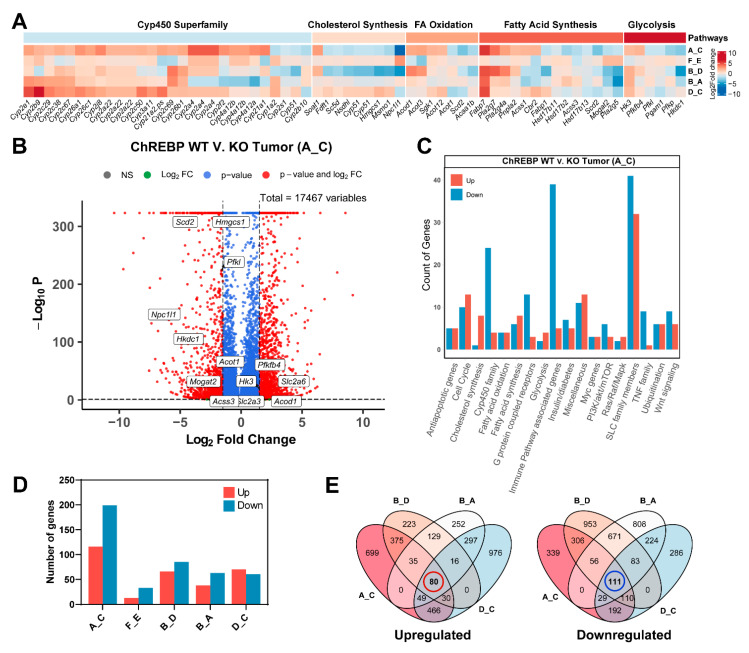
Systemic loss of ChREBP downregulates the expression of transcripts that encode enzymes involved in metabolic processes and vice versa. (**A**) A heat map representing altered significant metabolic genes involved in critical pathways of ChREBP^+/+^ WT and ChREBP^−/−^ tumors. Significance in up- and downregulation was calculated using log2 fold change >0.6 and *p*-value < 0.05. (**B**) An enhanced volcano plot comparing the differentially expressed genes and highlighted some dysregulated genes in total 17,467 variables between ChREBP WT and KO tumor. The plot represents the negative log10 of the *p*-value (Y-axis) and log2 of the fold change of gene expression (X-axis) for individual transcript of WT vs. KO tumor. The broken vertical lines represent fold change values of ±1.5. FC, fold change. (**C**) Bar plot showing dysregulated (up/down) genes of certain families (as in B) that function through mentioned processes. (**D**) Bar plot indicating the overall identified genes that are consistently dysregulated between groups. (**E**) Venn diagram demonstrating combined up- and downregulated genes when the comparison between A_C, B_D, B_A and D_C according to supplementary Figure S10 was performed. Shown in the red circle is the number of upregulated genes (80) and the number (111) in the blue circle represents downregulated gene numbers.

Given the role of ChREBP in promoting hepatocarcinogenesis, our RNA sequencing-based transcriptional profiling showed differentially expressed mRNA transcripts. Of a total of 17,467 genes, around 1777 genes were significantly upregulated by at least 1.5-fold (FDR < 0.05 and fold-change >= 1.5) in wild type tumor, whereas downregulated expression was noticed in 1222 genes in tumor that lacked ChREBP (supplementary Figure S8, group A_C). Among the differentially expressed genes, we manually sought and selected 315 interesting genes in consideration of their pivotal roles in metabolic pathways, and thus performed manual categorization according to their pathway regulation and contribution. Compared with the WT tumor, which presented upregulated expression in 199 genes, the tumor formed in KO mice displayed downregulation in 116 genes (Figure 6D). Although other groups exhibited differential gene numbers, in the group A_C (WT tumor vs. KO tumor) showed the genes with the highest differential expression, and group E_F (WT non-diabetic and KO non-diabetic control mice) displayed the lowest number of differentially expressed transcripts. Therefore, in the present study, we focused on identifying genes that exhibited consistent dysregulation between WT and KO tumor tissue.

As mentioned earlier, an intriguing characteristic of HCCs is their high regulation of glycolytic pathway [12]. It is noticeable from the results presented in Figure 6A that diabetes induced IPIT transplanted wild type tumor showed altered expression of certain significant genes associated with the glycolysis process. Gene *Pfkfb4,* with 1.7 fold upregulation in WT tumor, encodes the tissue specific 6-phosphofructo-2-kinase/fructose-2,6-bisphosphatase 4 enzyme and is considered to be activator of the key regulatory enzyme of the glycolysis, fructose 2,6-bisphosphate (F2,6BP) [25,26]. F2,6BP, in turn, allosterically activates the rate-limiting enzyme of 6-phosphofructo-1-kinase (PFK-1) in glycolysis process and its synthesis is reported to be highly stimulated in HCC by certain oncogenic alterations which presumably augment glucose consumption rate [27]. Besides *Pfkp* (2.8-fold decrease), which is a platelet-specific subunit of phosphofructokinase (PFK) enzyme, liver-specific PFK (*Pfkl*) also showed downregulation in their mRNA expression by 1.6-fold in KO mice relative to its corresponding WT mice. Decreased transcription (by 3.2-fold) of *Hkdc1* gene, a newly identified isoform of hexokinase, is evident in KO tumor as well. Previous research evidently showed hepatocyte specific high expression of *Hkdc1* is associated with poor prognosis in HCC [28].

Similarly, transcription of gene encoding hexokinase 3 (*Hk3*) was upregulated in tumor obtained from WT mice in comparison to ChREBP-KO tumor by a fold of 1.5. The sixth enzyme that displayed downregulated expression (1.6 fold decrease) in KO tumor is Pgam1. Notably, no genes presented significant changes in the expression of the above-mentioned enzymes between non-diabetic WT and KO control mice (Group F_E in Figure 6A,D).

It is widely accepted that sequential activation of glycolysis leads to induction of de novo lipogenesis and that deregulation in lipid biosynthesis is closely linked with HCC biological aggressiveness [29]. In line with this, we investigated whether hyperactive glycolysis leads to dysregulation in fatty acid synthesis and oxidation. We observed a significant number of genes including *Fabp7, Cbr2, Pla2g7, Pla2g4a, Pnpla2 and Acss1* were upregulated by an average fold of 2.7 in WT tumor, whereas transcription of *Scd2, Fabp1, pla2g5, Mogat2, Hsd17b2, Hsd17b11 and Hsd17b13* genes displayed an average 2.4-fold decrease in tumor that lacks ChREBP globally.

In addition, while four genes involved in fatty acid oxidation (FAO) exhibited a downregulation in their mRNA expression by an average fold of 2.4 in KO tumor, the rest genes showed upregulated expression by a factor of almost 2.0 in tumor formed in WT mice. Interestingly, we observed that mRNA levels of genes encoding cholesterol biosynthesis enzymes were mainly downregulated (mean fold change 2.7) in KO tumor relative to their corresponding WT tumor. Importantly, all mentioned genes pertaining to the various aforementioned processes have more or less previously been described as potential ChREBP targets [12]. We also sought the transcripts involved in the insulin signalling cascade that regulates glucose and lipid homeostasis. Results showed significant upregulated expression of certain genes like *Hgf, Hmga1, Rasgrp1, Sh2b2, Socs1, Socs2, and Socs3* in WT mice tumor compared to its ChREBP systemic knockout tumor.

The microsomal cytochrome P450 (CYP) families are the key players of fatty acid hydroxylation in human liver and kidney [30]. An analysis of genes pertaining to the cytochrome P450 superfamily showed a significant enrichment of P450 gene signature. Of 28 dysregulated genes, two dozen genes showed upregulation in their transcription by a mean fold of 2.3 in WT tumor tissue in comparison to ChREBP knock-out tumor.

Furthermore, significant cell cycle regulating genes belonging to cyclin-dependent kinases (Cdks), cell division cycles (Cdc genes) and anaphase-promoting complex/cyclosome (APC/C) that are known to play crucial role in cell cycle progression displayed differential regulation in their mRNA levels between tumor of WT and knock-out. Of these, polo-like kinase 1 (Plk1) showed 2.2-fold downregulated expression in ChREBP^−/−^ mice tumor. Previous research from our lab also convincingly showed upregulation of *Plk1* in ChREBP ^+/+^ mice tumor and a marked reduction in its mRNA level in mouse HCC cell line by concomitant ChREBP silencing [29].

Of at least 362 putative members of solute carrier (SLC) gene superfamily that are involved in transporting substrates especially glucose, amino acids and inorganic ions through membrane-bound transporters, dysregulated transcription of 76 genes was evident in both WT and KO tumor. Most genes belonged to the *Slc2, Slc5 and Slc37* family and function as sugar transporters. The *Slc2a* family, consisting of the genes *Slc2a3, Slc2a4*, *Slc2a5, Slc2a6 and Slc2a7*, is responsible for glucose transporters, and exhibited increased transcription in WT type mice tumor compared to KO tumor (Supplementary Figure S9).

We also investigated the transcriptional drivers that could prompt the enrichment of genes involved in numerous immunological processes. Considering a few exceptions for some genes, we detected average 3.7-, 2.4-, 3.5-, 2.4- and 2.5-fold increases in chemokine ligands transcripts (CCLs), chemokine receptors transcripts (CCRs), chemokine (C-X-C motif) ligand transcripts (CXCLs), transcripts encoding interleukins (ILs) and toll-like receptors (TLRs) transcripts, respectively. We analysed differentially expressed mRNA transcripts in tumor of WT mice in comparison to KO tumor and detected an average >2-fold increase in the myc gene family, anti-apoptotic genes and tumor necrosis factors (TNF).

While examining the mRNA levels of several key regulators of oncogenic signalling pathways, including Ras/Raf/Mapk, PI3K/AKT/mTOR and Wnt signalling, we found 5, 9 and 15 differentially expressed genes respectively between tumor of WT and KO. Tumors obtained from WT mice displayed upregulation in 13 transcripts (mean fold increase by 2.6) encoding G protein-coupled receptors, whereas KO tumor showed downregulated expression of three genes (mean fold decrease: 3.2). By comparing the gene expression between tumor and non-tumor liver tissue in WT and KO mice, we found few dysregulated genes that reached statistical significance.

Taken together, the bar plot in Figure 6C indicates the comparative pathway-specific involvement of highly and low regulated genes between the WT and KO tumor groups. Moreover, an elaborate comparison performed between A_C, D_C, B_A and B_D groups additionally showed upregulated expression of 80 significant genes in WT tumor and 111 downregulated in mice tumor with systemic knockout of ChREBP (Figure 6D).

Lastly, in an attempt to validate the results obtained from RNA-seq, we randomly selected a set of candidate genes specific for pathways involved in metabolism (Glucose transports, glycolysis, fatty acid synthesis and oncogene activation) and performed quantitative real-time PCR. Similar to RNA-seq data, gene expression analysis presented in supplementary Figure S9 show a similar trend in gene expression pattern for the representative transcripts.

Taken together, our study suggests that through altered expression of metabolic genes, certain pathways are highly regulated in HCCs and that is probably associated with elevated transcriptional signature of ChREBP.

**Figure 7:**
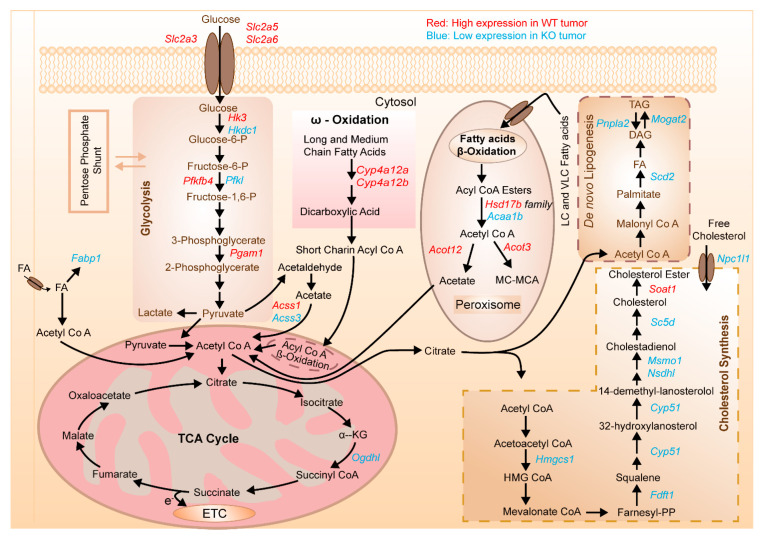
Schematic representation of the deregulated genes involved in different metabolic pathways in hormonally induced HCC. Enriched genes mentioned in each pathway are selected from heatmap (Figure 6A). Shown in red are the transcripts that displayed upregulated expression in tumors formed in wild type mice, while blue indicates downregulated genes in knockout tumor.

Based on the aforementioned RNA-seq-based gene expression data, we thus illustrated a simplified overview of certain metabolic pathways functioning through metabolism and highlighted the corresponding metabolic alterations pertinent to glycolysis, lipid metabolism and cholesterol synthesis due to marked mRNA upregulation and downregulation of particular genes through which hepatocarcinogenesis may arise. Figure 7 summarizes the mRNA transcripts whose expressions are significantly dysregulated in both WT and KO tumor.

## 4. Discussion

This is the first study proving the development of hepatocellular adenomas and carcinomas from glycogen-storing clear cell foci (CCF) in diabetic mice, both in wild type (WT) and ChREBP-knockout (KO) mice due to insulin-mediated metabolic and molecular alterations in hepatocytes after intraportal pancreatic islet transplantation. Thus, CCF are also pre-neoplastic in this mouse model, defining a suitable method to study hormonally induced hepatocarcinogenesis in the mouse, as it has previously only been demonstrated in diabetic rats [7,8]. Effects of local hyperinsulinism and the ensuing insulin-mimetic effects in altered hepatocytes of CCF have also been described in the rat previously by our group—including the translocation of the insulin receptor from the plasma membrane into the cytoplasm, an increased expression of the insulin receptor itself, and its downstream targets. Thus, severe alterations of insulin signalling were induced by local action of islet hormones in the liver and may substantially contribute to the carcinogenic process [9,12,31]. This is reinforced by the observation that genes (*Igfbp1 and Igfbp2*) encoding insulin like growth factor binding proteins and insulin-induced gene 1 (*Insig1*) were downregulated in tumor of KO mice. We also observed high expression of SLC genes involved in glucose transport in tumor obtained from WT mice (supplementary Figure S9).

In our previous short-term experiments [12], CCF in wild type mice was characterized by increased fat and glycogen accumulation, upregulation of glycolysis and de novo lipogenesis, increased proliferative activity and upregulation of the AKT/mTOR proto oncogenic pathway.

Glycolysis intensity is mainly regulated by the concerted actions of three physiologically irreversible enzymes: hexokinase, phosphofructokinase (Pfk-1), which is considered to be the gatekeeper of glycolysis, and a third enzyme, pyruvate kinase, a rate-limiting enzyme of glycolysis that shows dependence on ChREBP. At the onset of HCC, cancerous cells increase their metabolic output that result in enhanced rate of glycolysis and subsequent increase in de novo lipogenesis [12]. In line with this, our analyses convincingly showed an increase in several transcriptionally active genes that fuel the enzymes of glycolysis and fatty acid synthesis and oxidation in WT tumor (Figure 6A,B and supplementary Figure S9).

In contrast, CCF of ChREBP-knockout mice revealed no lipid droplets, but masses of glycogen due to decreased glycolysis, de novo lipogenesis, lower proliferative activity and downregulated AKT/mTOR signalling. In the current study, these alterations were also present which manifest hepatocellular carcinomas. Similarly, downregulated expression of genes involved in aforementioned pathways elicited comparatively delayed dynamics in those biological processes and thus led to the formation of delayed tumor in knock-out mice (Figure 7).

As already described, CCF in WT mice revealed a glycogenotic and lipogenic phenotype, which is a potential marker for further development to malignant hepatocellular tumors in other mouse models of hepatocarcinogenesis [13,31]. In contrast, deletion of ChREBP led to glycogenotic but not lipogenic CCF, with simultaneous lower proliferation. These findings suggest a correlation between ChREBP activity and the metabolic switch from a glycogenotic to a lipogenic phenotype and growth tendency in preneoplastic lesions in insulin related hepatocarcinogenesis. 

In the rat, this insulin-induced carcinogenesis model after IPIT is well established and assumed as a sequence of CCF to HCA and HCC [10,11,31]. However, in the mice model, development of HCA and HCC has not been described so far. HCCs occurred already after six months in WT mice, while tumors developed only at the end of 12 months in ChREBP-KO mice. 

The delayed carcinogenesis in KO mice could be the effect of downregulated AKT/mTOR signalling upon ChREBP depletion. Thus, the Warburg effect—activated by PI3K-AKT or Hypoxia inducible factor 1 (HIF1) as target genes of ChREBP—could partly be decreased in these mice [32,33]. Our gene expression analysis identified certain genes that are lowly expressed in KO tumor and their aberrant activation has pivotal roles in tumor progression.

Furthermore, a study by Iizuka et al. demonstrated a suppression of p53 and a switch from oxidative phosphorylation to aerobic glycolysis in cancer cells due to ChREBP induction [17]. Thus, ChREBP deletion could reduce the Warburg effect and enhance p53 activity, leading to inhibition of hepatocarcinogenesis in KO mice [24]. Even the occurrence of hepatocellular adenomas in diabetic, albeit not transplanted, WT mice and not in diabetic KO mice suggests a proto-oncogenic function of ChREBP in metabolic carcinogenesis in the liver. A proto-oncogenic potential of ChREBP in the liver could also be proven in the model of hydrodynamic gene transfer [29] with overexpression of AKT in ChREBP-knockout mice, leading to considerably less HCC frequency. 

In humans, ChREBP is also upregulated in proliferating glycogenotic liver foci, which resembles preneoplastic CCF of diabetic mice and rats [12]. Furthermore, an inverse correlation between survival of HCC patients and ChREBP expression could also be detected [29].

Our results demonstrate an important role of the transcription factor ChREBP in AKT/mTOR driven proliferation in hormonally induced CCF of altered hepatocytes in diabetic mice. ChREBP deletion seems to delay hepatocarcinogenesis and partly inverses AKT/mTOR related metabolic characteristics. 

Thus, elevated ChREBP could be a possible risk factor in human hepatocarcinogenesis, especially related to diabetes and NAFLD, and could also be a potential target in anti-tumoral therapy. 

In the near future, this will be the focus of further investigations of our research group, applying other hepatocarcinogenesis models, especially those associated with steatosis and steatohepatitis, to approximate the common human situation with slowly but steadily increasing hyperglycaemia and hyperinsulinism. In these future experiments, we assume a higher frequency of tumors, which will additionally be characterized at the genomic, epigenomic and proteomic level.

Proper elucidation of mechanistic differences between rapid tumor formation in WT mice and delayed tumor progression in ChREBP^−/−^ mice, together with proper identification of metabolic transcripts, will certainly help understand the underpinning mechanism of HCC development.

## 5. Conclusions

The pancreatic islet transplantation model is a suitable method for studying hormonally induced hepatocarcinogenesis also in mice, allowing a combination with gene knockout models. On the basis of RNA-transcriptome and immunohistochemistry analyses, we identified distinct differences in metabolic as well as in signalling pathways between tumors of ChREBP^+/+^ and ChREBP^−/−^ mice.

Our data indicate that deletion of ChREBP delays insulin-induced hepatocarcinogenesis, suggesting a combined oncogenic and lipogenic function of ChREBP along with AKT/mTOR-mediated proliferation of hepatocytes and induction of HCC.

## Figures and Tables

**Table 1:** Experimental and control groups.

	6 Months				12 Months			
	Transplantation		Control		Transplantation		Control	
	Diabetic	Nondiabetic	Diabetic	Nondiabetic	Diabetic	Nondiabetic	Diabetic	Nondiabetic
Wildtype C57Bl/6J	*n* = 36	*n* = 20	*n* = 20	*n* = 19	*n* = 33	*n* = 19	*n* = 13	*n* = 20
ChREBP-knockout	*n* = 18	*n* = 19	*n* = 18	*n* = 20	*n* = 12	*n* = 19	*n* = 13	*n* = 21

**Table 2:** Proliferation in normal liver tissue. Proliferative activity as BrdU-LI of normal liver parenchyma in wild type (WT) and ChREBP-knockout (KO) mice is shown. Comparison between WT and KO in each group.

Experimental Groups	6 Months (Mean ± S.E.M. (*n*))	12 Months (Mean ± S.E.M. (*n*))
WT diabetic transplanted	8.76 ± 0.90% (20) ^1^	10.63 ± 1.77% (20) ^2^
KO diabetic transplanted	5.16 ± 1.04% (9) ^1^	2.24 ± 0.61% (9) ^2^
WT transplanted	1.35 ± 0.27% (10)	1.49 ± 0.43% (10)
KO transplanted	6.10 ± 2.51% (10)	3.89 ± 2.33% (10)
WT diabetic	6.11 ± 2.18% (6)	2.57 ± 0.93% (9)
KO diabetic	2.11 ± 0.60% (9)	4.20 ± 1.31% (8)
WT non-diabetic	1.54 ± 0.33% (10)	1.03 ± 0.21% (10)
KO non-diabetic	2.21 ± 0.87% (10)	1.69 ± 0.47% (15)

^1^*p* < 0.05; ^2^ *p* < 0.001.

## Data Availability

All results generated or analyzed during present study are included in this published article and supplementary materials. Data and materials will be made available upon request via email to corresponding author.

## Supplementary Materials

The followings are available online at https://www.mdpi.com/article/10.3390/cells10102787/s1, Table S1: List of primary antibodies, Table S2: RNA concentration and quality measurement, Figure S1: Correlation in PAS quantification, Figure S2: Glycogen storage in CCF and extrafocal tissue, Figure S3: Proliferative activity in normal liver tissue after 6 and 12 months, Figure S4: Blood glucose levels after 6 and 12 months, Figure S5: Body weight after 6 and 12 months, Figure S6: Schematic illustration for NGS-based transcriptome experiment, Figure S7: Heat-maps representing dysregulated significant genes, Figure S8: Bar plots showing distinct deregulated (up/down) genes between groups, Figure S9: Validation of RNA-seq results by real-time RT-PCR, Figure S10: Quantification of serum ALT and AST, Figure S11: Representative histological images obtained from wild type and knock-out mice, Supplementary File 2, Script written for computer assisted PAS quantification, Supplementary File 3, Script for analyzing data of automated image analysis. Data files pertaining to NGS RNA-seq is available in NIH and can be retrieved with the BioProject id PRJNA771347. Data files pertaining to NGS RNA-seq is available in NIH and can be retrieved with the BioPro-ject id PRJNA772721 from SRA database and the release date is 2022-12-29. The SRA records will be accessible with the following link (https://www.ncbi.nlm.nih.gov/sra/PRJNA772721) after the indicated release date.
