# Supplementary material for: Hormonally Induced Hepatocellular Carcinoma in Diabetic Wild Type and Carbohydrate Responsive Element Binding Protein Knockout Mice
Source: Cells. 2021 Oct 18;10(10):2787. doi: 10.3390/cells10102787 (PMC8534692; doi:10.3390/cells10102787)
Supplement: Supplementary file 1 [file cells-10-02787-s001.zip › Supplementary File 1.pdf]

Hormonal induced hepatocellular carcinoma in diabetic Wild Type and Carbohydrate responsive element binding protein knockout mice

Vincent Nuernberger *et al*.

Supplementary materials and methods

Diabetes induction

The mice received a single intraperitoneal dose of streptozotocin (180 mg/kg body weight; Zanosar®, Sigma-Aldrich, Darmstadt, Germany). One week following induction, mice with a blood glucose level > 16.7 mmol/l were defined diabetic. After 1-week, pancreatic islet transplantation was performed.

Pancreatic Islet Transplantation

Pancreatic islets were extracted from donor mice from the same genotype. Mice were killed under anaesthesia (ketamine/xylazine 400/40 mg/kg body weight) and perfused with Neutral Red Solution. Pancreatic tissue was removed and mechanically cut with a razor blade. Exocrine tissue was digested with collagenase and albumin (2 mg and 1 mg per individual pancreas) and washed with HANK's solution (pH 7.2; Sigma-Aldrich, Darmstadt, Germany). Pure vital islets were collected manually with a pipette under a stereomicroscope and subsequently stored on ice.

Anesthetised (ketamine/xylazine 100/10 mg/kg body weight) recipient WT or ChREBP-KO mice received an intraportal transplantation of 200 isolated islets. For this scope, the portal vein was cannulised with a 26-gauge needle connected with a flexible tube system filled with pancreatic islets in 0.1 ml HANK's solution.

Continuous hyperglycaemia was maintained after pancreatic islet transplantation because of the low number of transplanted islets. Blood glucose level and body weight were measured on a regular basis following pancreatic transplantation.

Application of the nucleoside analog 5-Bromo-2'-deoxyuridine (BrdU)

One week before sacrifice, half of the animals were anaesthetised and osmotic mini pumps (Osmotic pump model 2001, Charles River Laboratories, Sulzfeld, Germany) filled with BrdU in sodium chloride solution (0.6 mg/d; Sigma-Aldrich, Darmstadt, Germany) were subcutaneously implanted between the scapulae.

Tissue processing

Animals were sacrificed under anaesthesia (ketamine/xylazine 400/40 mg/kg body weight) after 6 and 12 months. Tissue was perfused with a solution of 4 % dextran and 0.5 % procaine hydrochloride in Ringer solution (pH 7.4) via cannulisation of the aorta with a 23-gauge needle. The liver's middle lobe was clamped, removed and frozen in liquid nitrogen cooled isopentane (2-methyl-butane) as well as a piece of pancreatic tissue and the left kidney, respectively. The remaining tissue was perfused with a fixation mixture of 0.5 % glutaraldehyde, 3 % paraformaldehyde and 4 % dextran in Ringer solution (pH 7.4).

The liver was excised and cut into pieces of 1-2 mm thickness. Slices of liver, pancreatic tissue and kidney were embedded in paraffin as well as fixed in glutaraldehyde and embedded in glycid ether.

Paraffin slides of 1-2 µm thickness were used for staining by H&E and the periodic acid Schiff (PAS) reaction.

Immunohistochemistry

Formalin-fixed and paraffin-embedded serial liver sections in 1-2 µm thickness were manually stained for aldolase, hexokinase II, pyruvate kinase M2 (PKM2), phosphorylated/activated AKT (pAKT), mammalian target of rapamycin (mTOR), phosphorylated/activated ribosomal protein S6 (pRPS6), acetyl-CoA carboxylase (ACAC) and BrdU. For antigen retrieval, a citrate buffer of pH 6.0 was used. Endogenous peroxidase was cleared with 1 % hydrogen peroxide, and positive reactivity of primary antibodies was performed by the HRP polymer and DAB as the chromogen substrate (Dako, Glostrup, Denmark).

Fatty acid synthase (FASN) immunohistochemistry was conducted using an automated immunostainer (Leica Biosystems, Wetzlar, Germany) and a DAB kit.

Cryopreserved liver sections were manually stained for glucose transporter 4 (GLUT4), phosphofructokinase (PFKL) and glycogen synthase kinase 3ß (GSK-3ß).

The immunohistochemical reactions were assessed semi-quantitatively by comparing intensity in CCF or tumour with corresponding surrounding unaltered liver tissue. Negative controls were stained without any primary antibody.

All primary antibodies with detailed information are listed in Supplementary Table 1.

Supplementary Table 1. List of primary antibodies.

Protein	Antibody-ID	Host and Clonality	Dilution	Company
ACAC	3676S	rabbit monoclonal	1:100	CellSignaling
Aktin	A1978	mouse monoclonal	1:5000 ON	SIGMA
Aldolase	3188S	rabbit polyclonal	1:100 ON	CellSignaling
BrdU	M0744	mouse monoclonal	1:50 ON	Dako
FASN	610962	mouse monoclonal	1:100	BD BioSciences
GLUT4	ab654	rabbit polyclonal	1:100 ON	Abcam
GSK-3ß	9323S	rabbit monoclonal	1:100 ON	CellSignaling
Hexokinase II	bs9455R	rabbit polyclonal	1:300 ON	Bioss Antibodies
p-AKT	4691S	rabbit monoclonal	1:100	CellSignaling
PFKL	ab37583	rabbit polyclonal	1:100	Abcam
PKM2	4053S	rabbit monoclonal	1:100 ON	CellSignaling
p-mTOR	2976L	rabbit monoclonal	1:100	CellSignaling
p-RPS6	2211S	rabbit polyclonal	1:100	CellSignaling

ON: overnight incubation; IHC: immunohistochemistry; WB: western blot

CellSignaling, Frankfurt/Main, Germany
Dako, Glostrup, Denmark
BD BioSciences, Heidelberg, Germany
Abcam, Cambridge, UK
Bioss Antibodies, Wobum, USA
Santa Cruz Biotechnologies, Heidelberg, Germany
Sigma-Aldrich, Darmstadt, Germany
Peter Roach and Dyann Segvich, Indianapolis, USA

Supplementary Table 2. RNA concentration (ng/µL) and quality (RQN value) as measured by Nanodrop and Bioanalyser respectively.

Sample No	Sample Name	Grouping for NGS analysis	Conc. (ng/µL)	RQN value
1	6851-T	**A**	471,8	9.4
2	6851-NT	**B**	510,3	7
3	6889-T	**C**	538,3	8.9
4	6889-NT	**D**	493,9	6.8
5	6589-K	**E**	476,8	6.7
6	6590-K	**E**	772,8	7.7
7	6544-K	**F**	603,7	9
8	6545-K	**F**	466,5	8.5

T=Tumor, NT= Non-tumor, K= Control

Glycogen quantification (automated PAS-quantification)

Paraffin-embedded liver sections were stained by the PAS reaction and counterstained with haematoxylin in a Leica immunostainer. Sections were visually evaluated and imaged on a DMRB microscope (Leica, Wetzlar, Germany) using a DS-Fi1 digital camera (Nikon Instruments, Düsseldorf, Germany) controlled by the NIS Elements BR software. For automated PAS-quantification adapted from Schaart *et al* *[1]*, four images of representative areas of each liver section were acquired using identical manual acquisition setting (same gain, exposure time, brightness, aperture and objective). Using Fiji (version 2.0.0 ImageJ), images were automatically analysed through a macro (supplementary file S2). It consists of the following steps: colour deconvolution (colour deconvolution-vectors manually determined from a representative image using the colour deconvolution by ROI option on areas with strong PAS stain and no PAS stain were applied to all images), measurement of mean colour intensities and "PAS"-stained areas in the images representing PAS staining. Using full colour images and global threshold, the area covered by tissue was determined for the use as a normalizer. Data generated by this automated image analysis was analysed using R (version 3.5.1; supplementary file S3) [2,3]. In brief, mean and standard deviation of "intensities of PAS stain" as well as "area of PAS stained pixels" and "area of tissue pixels" were calculated per each liver section. Percentage of PAS stained pixels in tissue pixels resulted in our PAS quantification as “Proportion of PAS positive pixels”. We opted for this area adapted proportion because of a better correlation between our calculation and visual evaluation of PAS stained liver sections as measured PAS intensities (supplementary file 2). Of 34 liver samples (18 diabetic wild type, 15 non-diabetic wild type, 1 non-diabetic ChREBP-KO mice) with PAS intensities covering the full range from weakly stained to strongly stained, glycogen content was determined biochemically using the Glycogen Assay Kit II (ab169558; Abcam, Cambridge, UK), according to the manufacturer's protocol to measure glycogen.

Biochemical Assays for measuring serum parameters

Alanine aminotransferase (ALT) and aspartate aminotransferase (AST) level in mice serum was assessed using alanine aminotransferase activity assay kit (cat. No: MAK055) and aspartate aminotransferase activity assay kit (cat. No: MAK052) (sigma-aldrich), respectively, using manufacturer’s protocol. Colorimetric read for each assay was determined using a commercially available automated colorimetric system (BMG labtech).

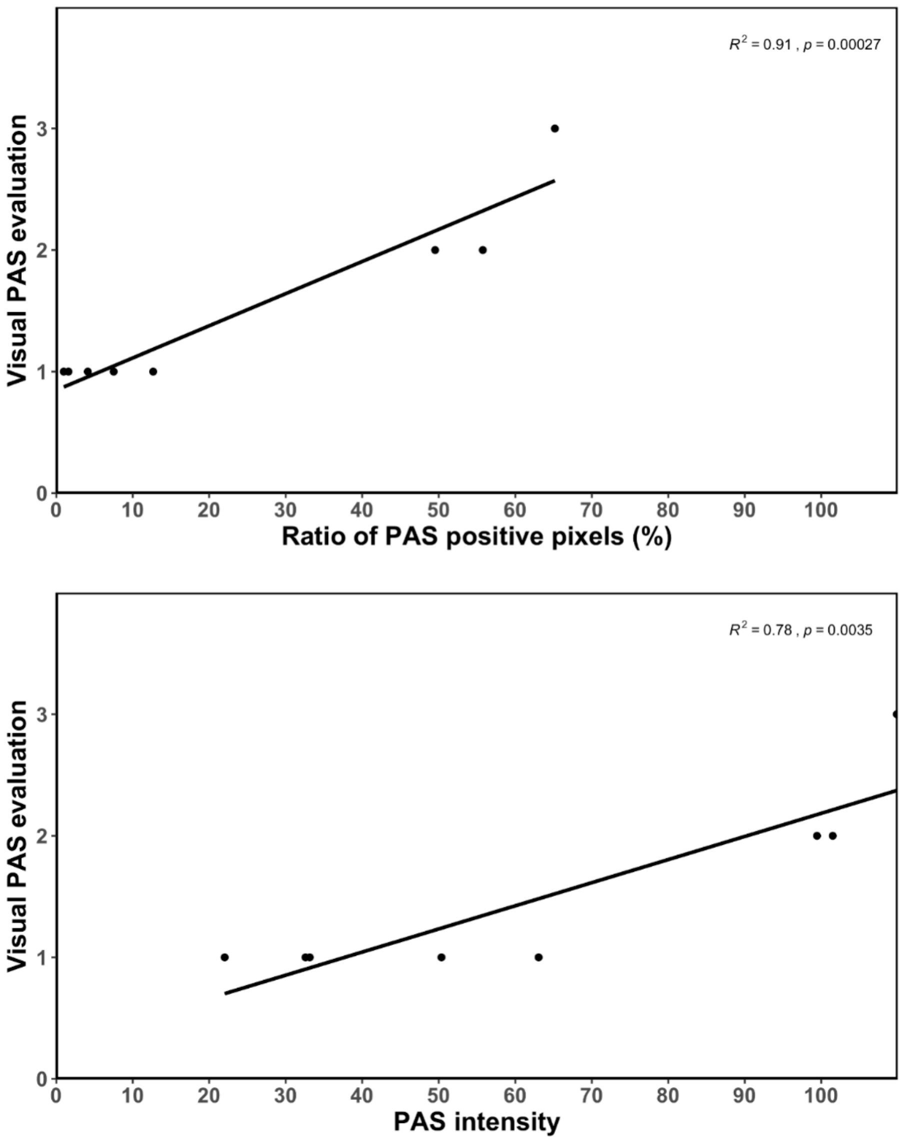

Supplementary Figure S1. Correlation in PAS quantification.

Figure shows correlation between visual PAS evaluation and measured proportion of PAS positive pixels as well as PAS intensity. Visual evaluation denotes three categories: strong PAS reaction (3 points), mediate reaction (2 points) and weak reaction (1 point). There was a significant correlation between visual evaluation and measured proportion of PAS positive pixels. Therefore, we decided to use this measurement for PAS quantification.

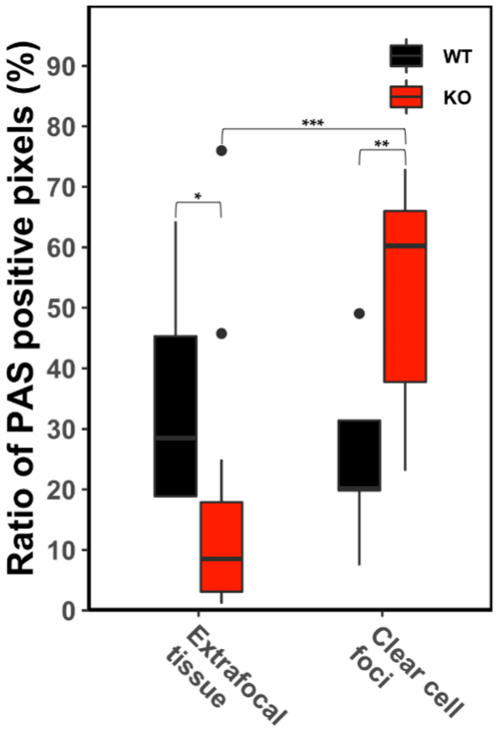

Supplementary Figure S2. Glycogen storage in CCF and extrafocal tissue.
Box plot depicts glycogen storage as proportion of PAS positive pixels in CCF as well as in extrafocal tissue of diabetic transplanted wild type (WT) and ChREBP-knockout mice (KO). KO mice stored more glycogen in CCF than in the extrafocal tissue. Similarly, CCF in KO mice revealed more glycogen than CCF in WT mice. Conversely, normal liver tissue of diabetic transplanted WT mice stored more glycogen than the tissue of KO mice. * $p < 0.05$; ** $p < 0.01$; *** $p < 0.001$.

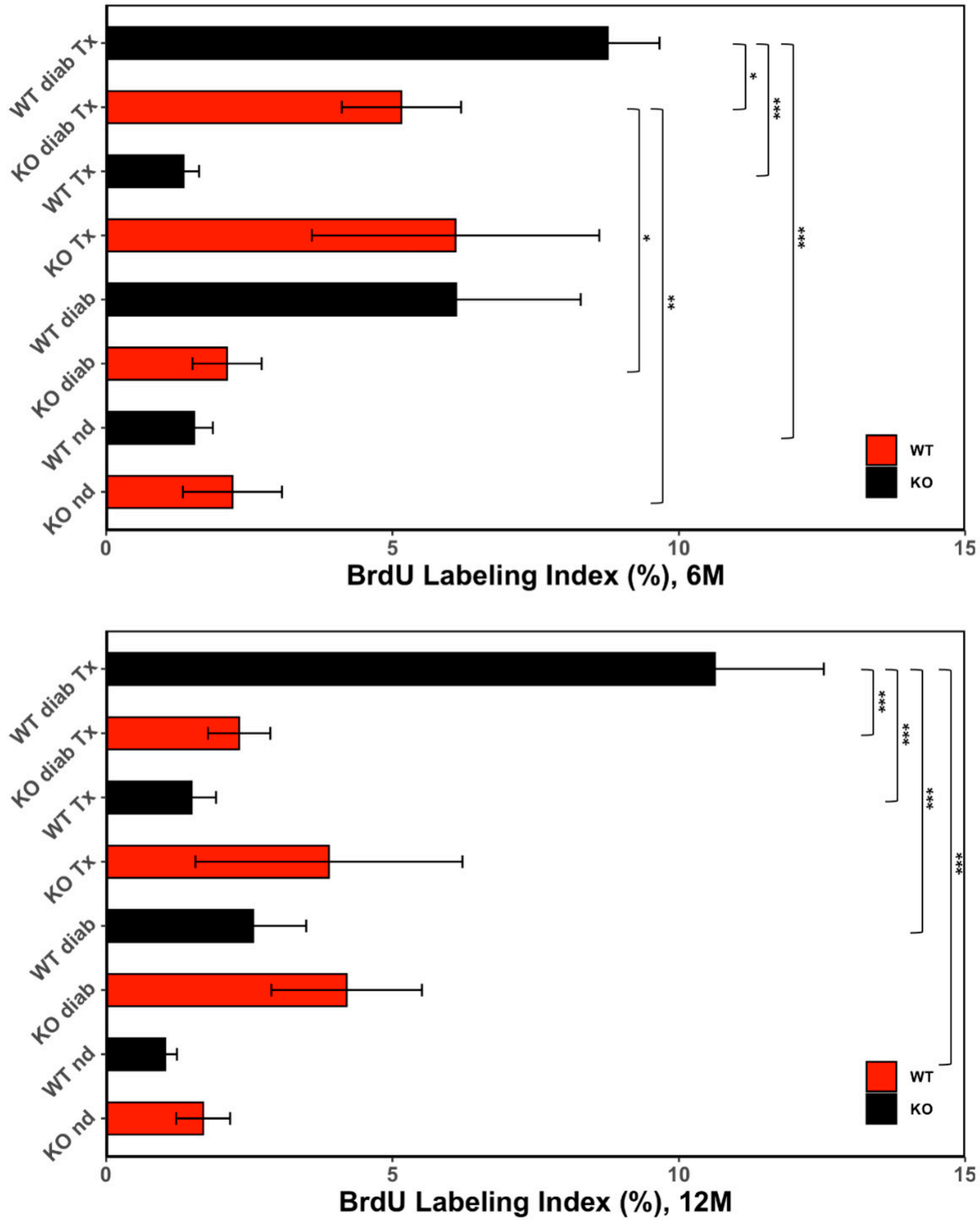

Supplementary Figure S3. Proliferative activity in normal liver tissue after 6 and 12 months.
Proliferative activity of extrafocal liver tissue as BrdU Labelling Index of non-diabetic (nd), diabetic (diab), non-diabetic transplanted (Tx) and diabetic transplanted (diab Tx) wild type (WT) and ChREBP-knockout mice (KO) after 6 (upper panel) and 12 months (lower panel). Diabetic transplanted WT mice revealed a significant higher proliferation than KO mice after 6 as well as 12 months. Furthermore, there were differences between the groups in each strain. Diabetic transplanted mice appeared to have a higher proliferative activity than control mice. * $p < 0.05$; ** $p < 0.01$; *** $p < 0.001$.

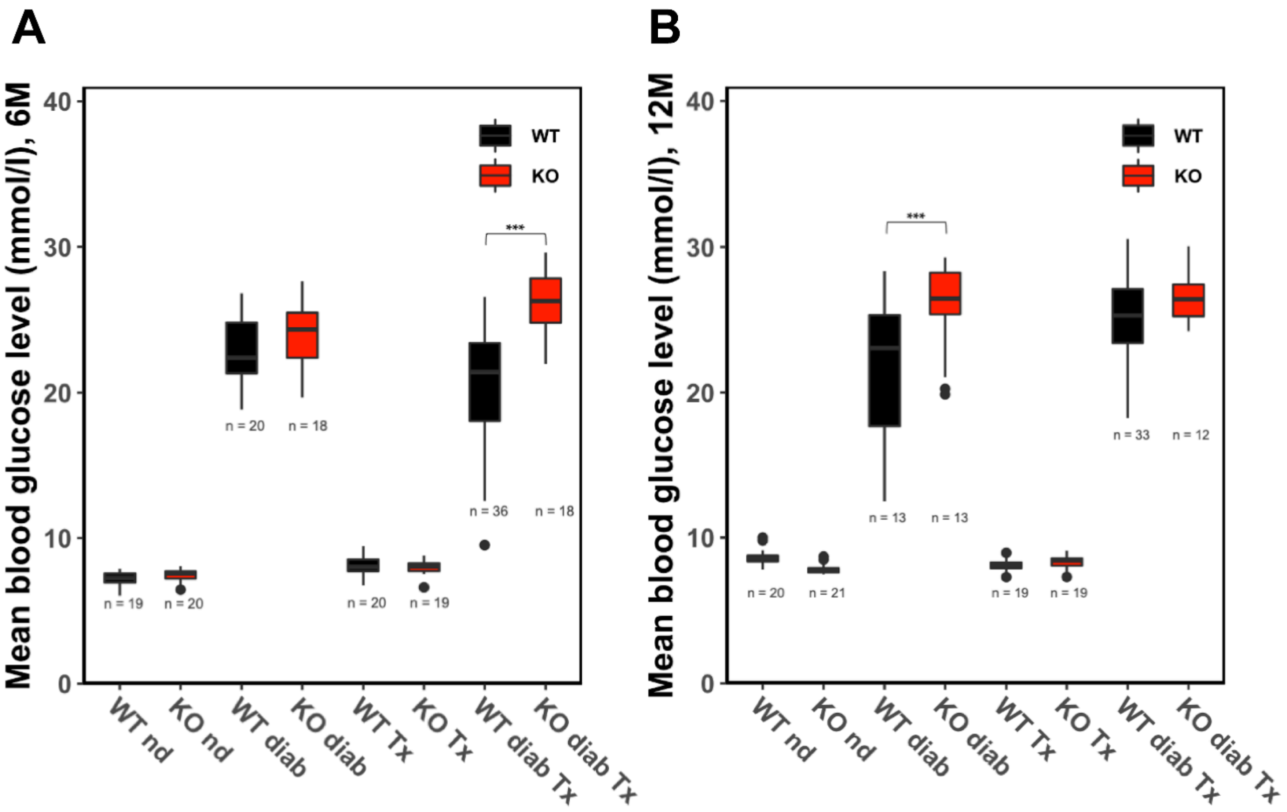

Supplementary Figure S4. Blood glucose levels after 6 and 12 months.

Blood glucose level of non-diabetic (nd), diabetic (diab), non-diabetic transplanted (Tx) and diabetic transplanted (diab Tx) wild type (WT) and ChREBP-knockout mice (KO) after 6 (A) and 12 months (B). No variation, independent of pancreatic islet transplantation, was found in non-diabetic mice. Diabetic transplanted WT mice revealed less blood glucose than diabetic transplanted KO mice after 6 months, a similar observation was also noticed in 12 months diabetic WT and KO mice. ** $p < 0.01$; *** $p < 0.001$.

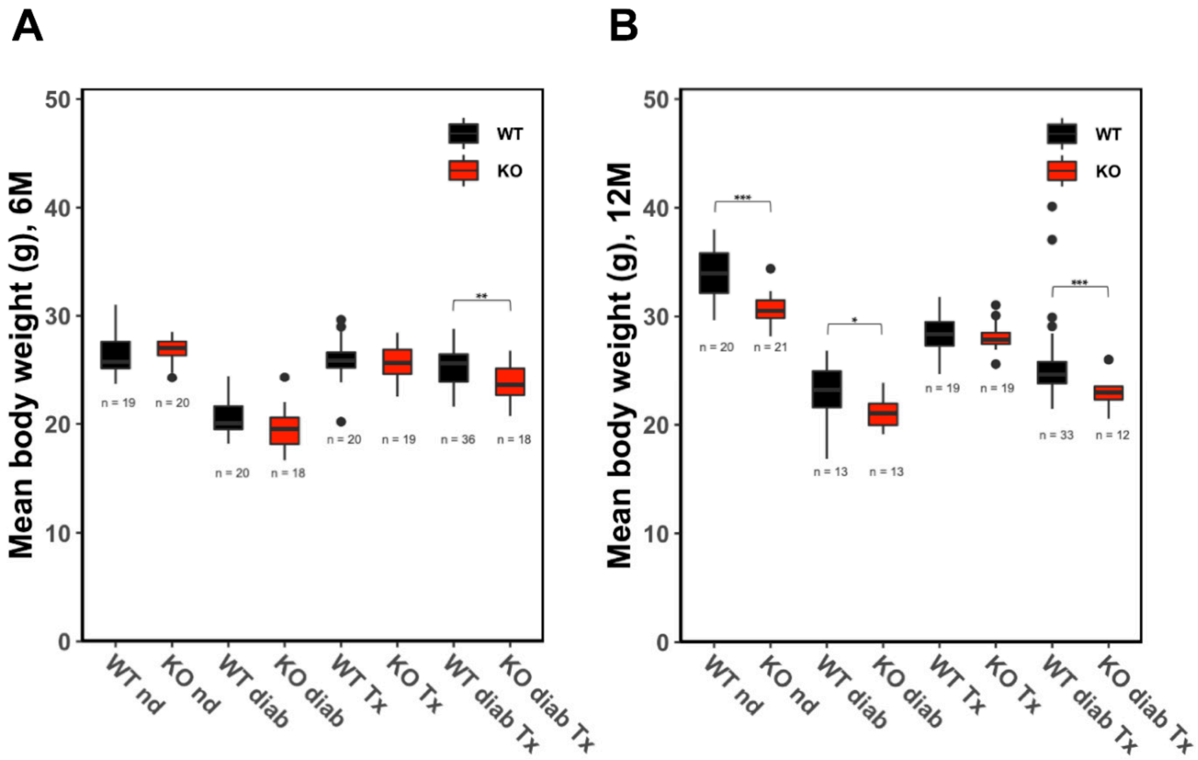

Supplementary Figure S5. Body weight after 6 and 12 months.
Body weight of non-diabetic (nd), diabetic (diab), non-diabetic transplanted (Tx) and diabetic transplanted (diab Tx) wild type (WT) and ChREBP-knockout mice (KO) after 6 (A) and 12 months (B). Diabetic transplanted KO mice had a lower body weight than diabetic transplanted WT mice after 6 and 12 months. Also, diabetic and non-diabetic WT mice revealed a higher body weight than KO mice after 12 months. * $p < 0.05$; ** $p < 0.01$; *** $p < 0.001$.

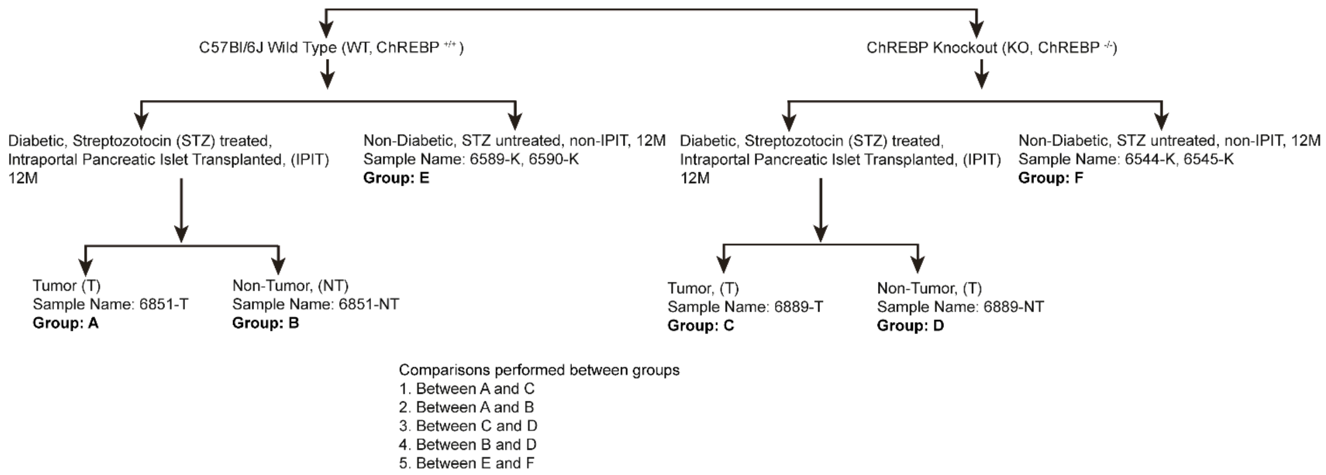

Supplementary Figure S6. This schematic illustration indicates the outline of comparisons performed between the groups for NGS-based transcriptome analysis. Tumor formed in each WT and KO mice was compared in between and also with the matched non-tumor tissue.

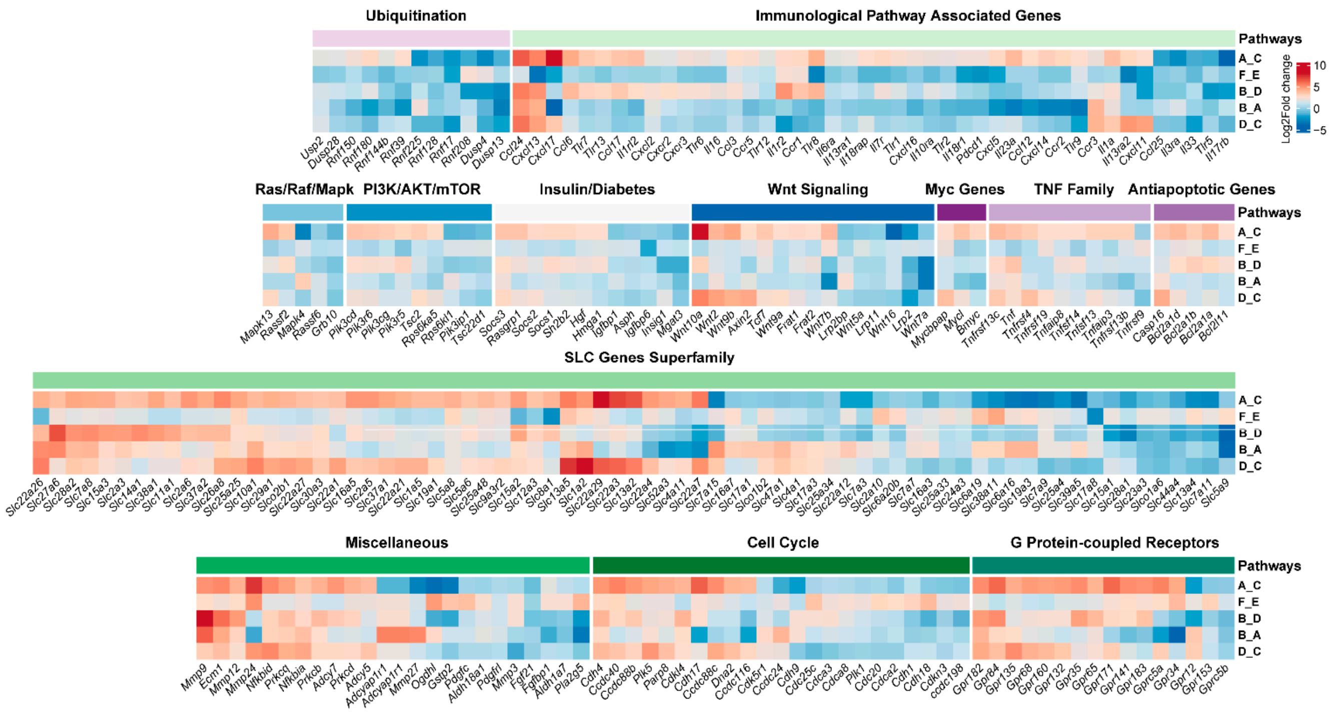

Supplementary Figure S7. Heat-maps representing dysregulated significant genes associated with critical pathways of ChREBP$^{+/+}$ WT and ChREBP$^{-/-}$ tumors according to Supplementary Figure S6. Significance in up and down regulation was calculated using log2 fold change > 0.6 and p-value < 0.05.

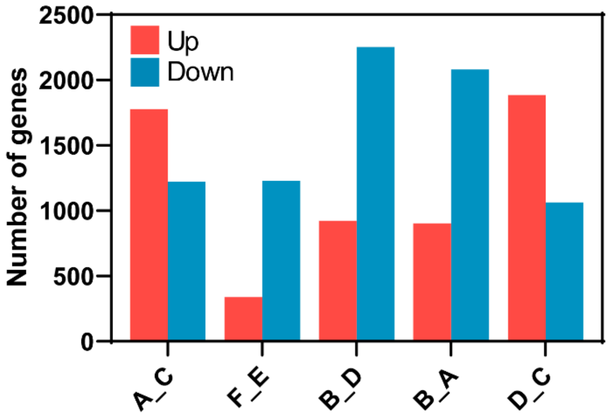

Supplementary Figure S8. Bar plots showing distinct deregulated (up/down) genes between groups as mentioned in the supplementary Figure S6. Shown are the gene numbers that displayed differential expression and reached statistical significance at least 1.5-fold (FDR <= 0.05 and fold-change >= ±1.5).

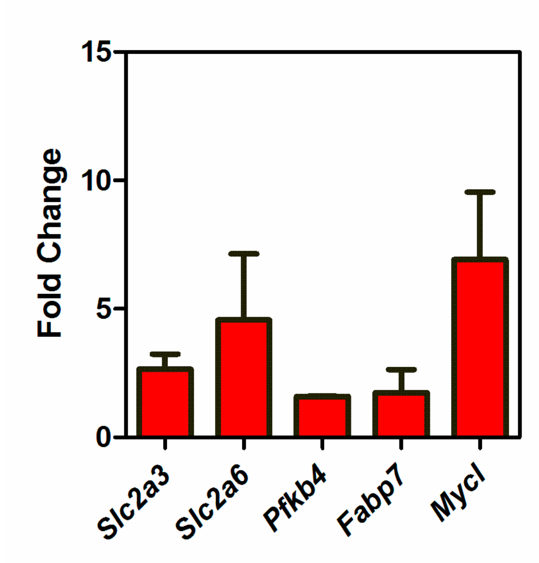

Supplementary Figure S9. Validation of RNA-seq results by real-time RT-PCR. Quantitative gene expression data analysis was performed by comparing gene of interest relative to housekeeping control gene (18S) between tumor groups (wild type and knock-out) with corresponding non-tumor sample and performed according to $2^{-\Delta\Delta CT}$ method [4]. Presented results show the mean and standard deviation (SD) from two independent experiments.

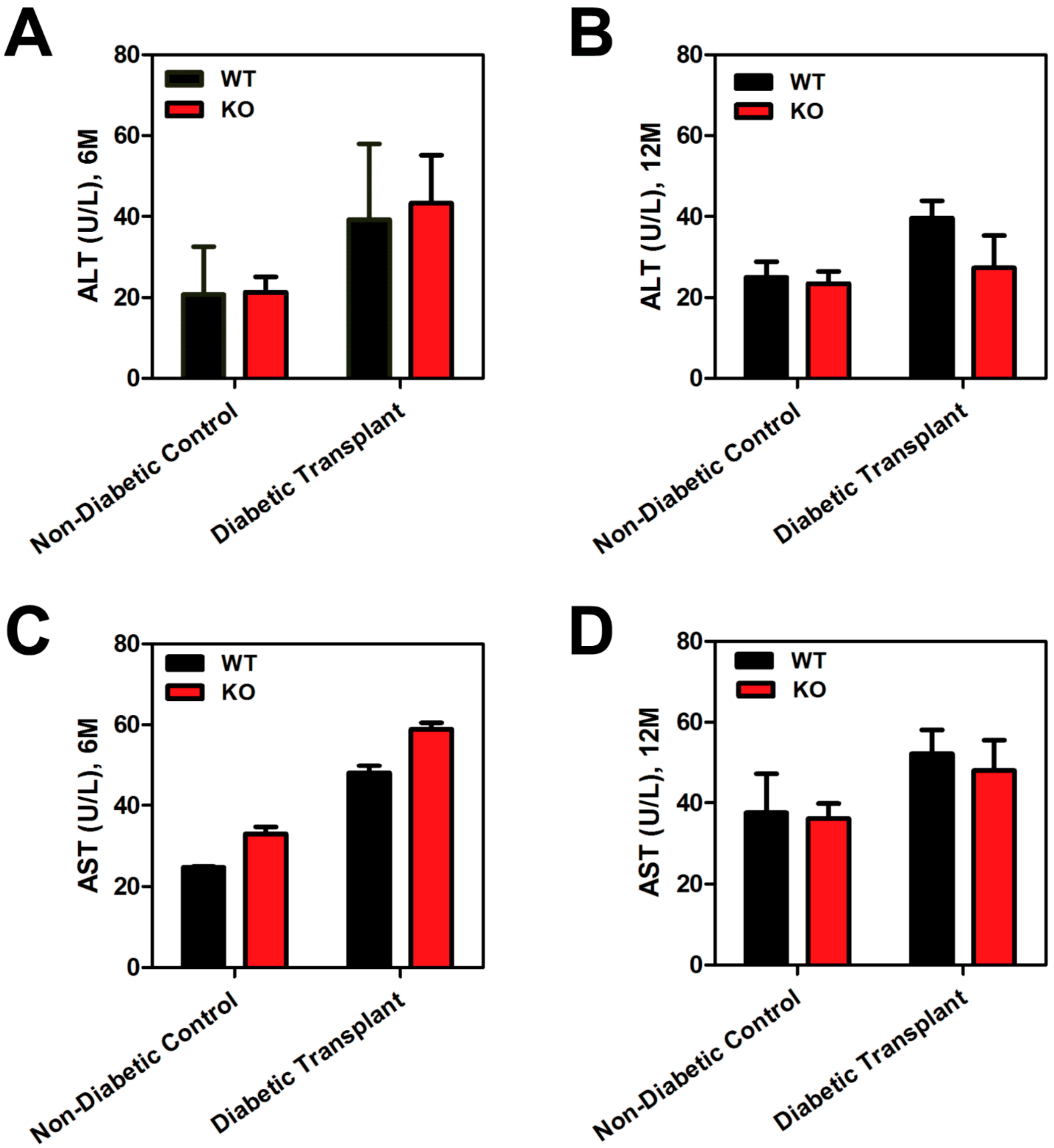

Supplementary Figure S10. Quantification of serum Alanine aminotransferase (ALT; upper panel) and aspartate aminotransferase (AST; lower panel) level in wild type (WT) and knockout (KO) mice (6 and 12 months). Obtained results are presented as mean ± SEM (n ≥ 5).

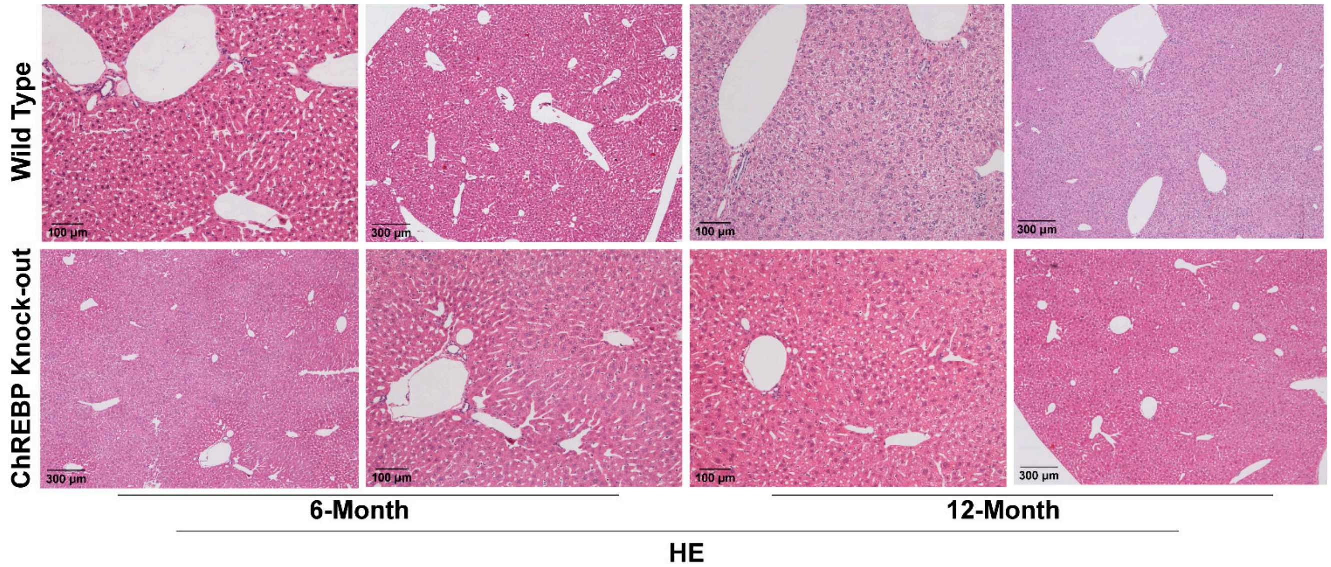

Supplementary Figure S11. Representative histological images obtained from wild type (WT) and knock-out (KO) mice (6 and 12 months) showing no visible signs of inflammation and cirrhosis.

Supplementary References

1. Schaart, G.; Hesselink, R.P.; Keizer, H.A.; van Kranenburg, G.; Drost, M.R.; Hesselink, M.K. A modified PAS stain combined with immunofluorescence for quantitative analyses of glycogen in muscle sections. *Histochemistry and cell biology* **2004**, *122*, 161-169.
2. Schindelin, J.; Arganda-Carreras, I.; Frise, E.; Kaynig, V.; Longair, M.; Pietzsch, T.; Preibisch, S.; Rueden, C. 714 Saalfeld S, Schmid B, Tinevez JY, White DJ, Hartenstein V, Eliceiri K, Tomancak P, Cardona A. 2012. 715 Fiji: an open-source platform for biological-image analysis. *Nat Methods 9*, 676-682.
3. Team, R.C. R: A language and environment for statistical computing. **2018**.
4. Schmittgen, T.D.; Livak, K.J. Analyzing real-time PCR data by the comparative CT method. *Nature protocols* **2008**, *3*, 1101-1108.
