# Supplementary material for: Hormonally Induced Hepatocellular Carcinoma in Diabetic Wild Type and Carbohydrate Responsive Element Binding Protein Knockout Mice
Source: Cells. 2021 Oct 18;10(10):2787. doi: 10.3390/cells10102787 (PMC8534692; doi:10.3390/cells10102787)
Supplement: Supplementary file 1 [file cells-10-02787-s001.zip › Supplementary File 2.pdf]

Script written for computer assisted PAS quantification.

The following script was written in Fiji (ImageJ) for the computer assisted PAS quantification.

```
macro "H&PAS color deconvolution" {
input = getDirectory("Input directory");
output = getDirectory("Output directory");
 Dialog.create("File type");
Dialog.addString("File suffix: ", ".jpg", 5);
Dialog.show();
suffix = Dialog.getString();
 processFolder(input);
 function processFolder(input) {
    list = getFileList(input);
    for (i = 0; i < list.length; i++) {
        if(File.isDirectory(list[i]))
            processFolder("" + input + list[i]);
        if(endsWith(list[i], suffix))
            processFile(input, output, list[i]);
    }
}
 function processFile(input, output, file) {
    print("Processing: " + input + file);
    open(input + file);
    title = getTitle();
            directory = File.directory();
            write("processing: "+title+" @"+directory);
            run("Colour Deconvolution", "vectors=[H PAS] hide");
            selectWindow(title+"-(Colour_3)");
            close();
            selectWindow(title+"-(Colour_2)");
            run("Measure");
            saveAs("TIFF", output+title+"-colour_2");
            //setThreshold(0, 150);
            setThreshold(0, 150);
            setOption("BlackBackground", false);
            run("Convert to Mask");
            run("Measure");
            saveAs("JPEG", output+title+"-colour_2_mask");
            close();
            selectWindow(title+"-(Colour_1)");
            run("Measure");
            saveAs("TIFF", output+title+"-colour_1");
            close();
            selectWindow(title);
            run("8-bit");
            //setThreshold(0, 200);
            setThreshold(0, 200);
            setOption("BlackBackground", false);
            run("Convert to Mask");
            run("Measure");
            saveAs("JPEG", output+title+"_mask");
    close();
}
}
```
