# Supplementary material for: Hormonally Induced Hepatocellular Carcinoma in Diabetic Wild Type and Carbohydrate Responsive Element Binding Protein Knockout Mice
Source: Cells. 2021 Oct 18;10(10):2787. doi: 10.3390/cells10102787 (PMC8534692; doi:10.3390/cells10102787)
Supplement: Supplementary file 1 [file cells-10-02787-s001.zip › Supplementary File 3.pdf]

R script for analyzing data of automated image analysis.

```
## loading results of Fiji analysis
Frame <- read.table("Results.csv", header = TRUE, sep = ",", quote = "")
Frame$imageType <- c("PASint", "PASarea", "H_int", "tissueArea")
## create dataframe with all relevant data
PAS <- data.frame(mouseID = substring(Frame$Label[Frame$imageType=="PASint"], 1,4),
        mouseGenotype = substring(Frame$Label[Frame$imageType=="PASint"], 10,11),
        mouseTransplantation = substring(Frame$Label[Frame$imageType=="PASint"], 16,19),
        mouseDiabetic = substring(Frame$Label[Frame$imageType=="PASint"], 21,24),
        mouseAge = substring(Frame$Label[Frame$imageType=="PASint"], 13,14),
        CCF = substring(Frame$Label[Frame$imageType=="PASint"], 26,29),
        Tumor = substring(Frame$Label[Frame$imageType=="PASint"], 31,34),
        ImageID = substring(Frame$Label[Frame$imageType=="PASint"], 6,6),
        PASvisual = substring(Frame$Label[Frame$imageType=="PASint"], 8,8),
        PASintensity = 255-Frame$Mean[Frame$imageType=="PASint"],
        ImageArea = Frame$Area[Frame$imageType=="PASarea"],
        PASArea = Frame$X.Area[Frame$imageType=="PASarea"],
        H_Intensity = 255-Frame$Mean[Frame$imageType=="H_int"],
        TissueArea = Frame$X.Area[Frame$imageType=="tissueArea"])
## count pixels of tissue
PAS$tissuePixels <- PAS$ImageArea*PAS$TissueArea/100
## count pixels of PAS
PAS$PASPixels <- PAS$ImageArea*PAS$PASArea/100
## count PAS/tissue x 100 -> PAS in %
PAS$PASquant <- PAS$PASPixels/PAS$tissuePixels*100

## summarise PAS
library(plyr)
PAS_sum <- ddply(PAS, ~mouseID, summarise, meanPASArea=mean(PASArea), sdPASArea=sd(PASArea), meanTissueArea=mean(TissueArea), sdTissueArea=sd(TissueArea), meantissuePixels=mean(tissuePixels), sdtissuePixels=sd(tissuePixels), meanPASPixels=mean(PASPixels), sdPASPixels=sd(PASPixels), meanPASquant=mean(PASquant), sdPASquant=sd(PASquant), meanPASintensity=mean(PASintensity), sdPASintensity=sd(PASintensity))
## remove unique data
PAS_unique <- subset(PAS, select = c("mouseID", "mouseGenotype", "mouseTransplantation", "mouseDiabetic", "mouseAge", "CCF", "Tumor", "PASvisual"))
## remove redundant data
PAS_unique <- PAS_unique[!duplicated(PAS_unique),]
## merge PAS_unique and PAS_sum
PASframe <- merge(PAS_unique, PAS_sum, by="mouseID")

## delete all other frames
rm(Frame)
rm(PAS)
rm(PAS_sum)
rm(PAS_unique)
```
